# Loss of *Phd2* cooperates with *BRAF*^*V600E*^ to drive melanomagenesis

**DOI:** 10.1038/s41467-018-07126-9

**Published:** 2018-12-21

**Authors:** Shujing Liu, Gao Zhang, Jianping Guo, Xiang Chen, Jingce Lei, Kan Ze, Liyun Dong, Xiangpeng Dai, Yang Gao, Daisheng Song, Brett L. Ecker, Ruifeng Yang, Caitlin Feltcher, Kai Peng, Cheng Feng, Hui Chen, Rebecca X. Lee, Heddy Kerestes, Jingwen Niu, Suresh Kumar, Weiting Xu, Jie Zhang, Zhi Wei, James S. Martin, Xiaoming Liu, Gordon Mills, Yiling Lu, Wei Guo, Lunquan Sun, Lin Zhang, Ashani Weeraratna, Meenhard Herlyn, Wenyi Wei, Frank S. Lee, Xiaowei Xu

**Affiliations:** 1 Department of Pathology and Laboratory Medicine, Perelman School of Medicine, University of Pennsylvania, Philadelphia, PA 19104 USA; 2 The Wistar Institute, Philadelphia, PA 19104 USA; 3 Department of Pathology, Beth Israel Deaconess Medical Center, Harvard Medical School, Boston, MA 02115 USA; 4 Xiangya Hospital, Central South University, Changsha, 410008 China; 5 Department of Computer Science, New Jersey Institute of Technology, Newark, NJ 07102 USA; 6 Department of Systems Biology, The University of Texas MD Anderson Cancer Center, Houston, TX 77030 USA; 7 Department of Biology, School of Arts and Sciences, University of Pennsylvania, Philadelphia, PA 19104 USA; 8 Center for Research on Reproduction & Women’s Health, University of Pennsylvania, Philadelphia, PA 19104 USA

## Abstract

Prolyl hydroxylase domain protein 2 (PHD2) is a well-known master oxygen sensor. However, the role of PHD2 in tumor initiation remains controversial. We find that during the transition of human nevi to melanoma, the expression of PHD2 protein is significantly decreased and lower expression *PHD2* in melanoma is associated with worse clinical outcome. Knockdown of *PHD2* leads to elevated Akt phosphorylation in human melanocytes. Mice with conditional melanocyte-specific expression of *Phd2*^*lox/lox*^ (*Tyr::CreER;Phd2*^*lox/lox*^) fail to develop pigmented lesions. However, deletion of *Phd2* in combination with expression of *BRaf*^*V600E*^ in melanocytes (*Tyr::CreER;Phd2*^*lox/lox*^*;BRaf*^*CA*^) leads to the development of melanoma with 100% penetrance and frequent lymph node metastasis. Analysis of tumor tissues using reverse phase protein arrays demonstrates that *Phd2* deletion activates the AKT-mTOR-S6 signaling axis in the recovered tumors. These data indicate that PHD2 is capable of suppressing tumor initiation largely mediated through inhibiting of the Akt-mTOR signaling pathway in the melanocyte lineage.

## Abstract (web summary)

Prolyl hydroxylase domain protein 2 (PHD2) regulates cellular response to hypoxia. Here the authors show that PHD2 is downregulated in melanoma and that PHD2 depletion, in a mouse model, promotes the progression of benign melanocytic lesions into melanoma, via activation of the Akt/mTOR signaling cascade.

## Introduction

Prolyl hydroxylase domain proteins (PHDs), also referred to as hypoxia-inducible factor (HIF) prolyl hydroxylases or EGLNs, form an evolutionarily conserved subfamily of dioxygenases that hydroxylate HIFs within the oxygen-dependent degradation (ODD) domains^1–3^. Three mammalian PHD isoforms, PHD1, PHD2 and PHD3, demonstrate distinct tissue-specific expression patterns and cellular function^4,5^. Among three PHD isoforms, PHD2, also known as EGLN1, is a 46 kDa protein enzyme that consists of an N-terminal domain homologous to MYND zinc finger domains, and a C-terminal domain homologous to the 2-oxoglutarate family of dioxygenases. PHD2 is considered the master “oxygen sensor” in a cell, and PHD2 regulates the hypoxia-induced gene transcription program largely by controlling the stability of HIF-α through prolyl hydroxylation in a site-specific manner^6,7^. Prolyl hydroxylated HIF-α components are recognized by the von Hippel–Lindau protein (pVHL) which mediates their polyubiquitination with subsequent proteasomal degradation.

Melanocytes are more prone to oncogenic transformation when grown in a hypoxic microenvironment, and this is in part due to stabilization of HIF-1α, because HIF-1α-deficient melanocytes grown in hypoxic conditions show a diminished transformation capacity and delayed tumor growth in vivo. On the other hand, the expression of an non-degradable form of HIF-1α protein leads to transformation of cells in normoxic conditions and to the growth of very aggressive melanomas^8^. Hypoxia is a common feature in solid tumors including melanoma, and hypoxia promotes melanoma progression^8,9^. Loss of PHD2 function leads to increased HIF-1α activity, increased glycolysis and improved adaptation to hypoxia^10,11^. We and others showed that mutations in the human *PHD2* gene are associated with erythrocytosis^12–14^. Loss of heterozygosity of *PHD2* in paraganglioma has been reported and suggests that *PHD2* can function like a tumor suppressor gene^12^. Nevertheless, to date, the role of PHD2 in cancer progression remains controversial. It has been shown that *PHD2* knockdown in breast cancer cells reduced the growth of xenograft tumors^15^, and that global *Phd2* haplo-deficiency impairs tumor invasion and metastasis^7,16^. On the other hand, in vivo xenograft models supported a tumor-suppressing role of PHD2 in pancreatic cancer^17,18^.

More recently, prolyl hydroxylase inhibitors (PHIs), such as roxadustat and produstat, are been tested in phase III clinically trials in patients with anemia (ClinicalTrials.gov). PHIs stimulate synthesis of renal and hepatic erythropoietin and improve hematocrit as well as stimulate neoangiogenesis^19,20^. Although PHIs appear to have a good safety profile in early phase clinical trials, long-term studies have not been performed. One significant concern regarding the long-term use of these agents is their possible effect on tumor growth^21^.

Here we show that melanocyte-specific deletion of *Phd2* cooperates with *BRaf*^*CA*^ to induce melanomagenesis. Haplo-deficiency of *Phd2* is also able to induce melanomagenesis despite a weaker effect. *Phd2* deletion activates the AKT–mammalian target of rapamycin (mTOR) signaling axis in tumor cells. These data support that PHD2 acts as a tumor suppressor in melanocytic tumors.

## Results

### PHD2 expression and function in human melanomas

**Fig. 1:**
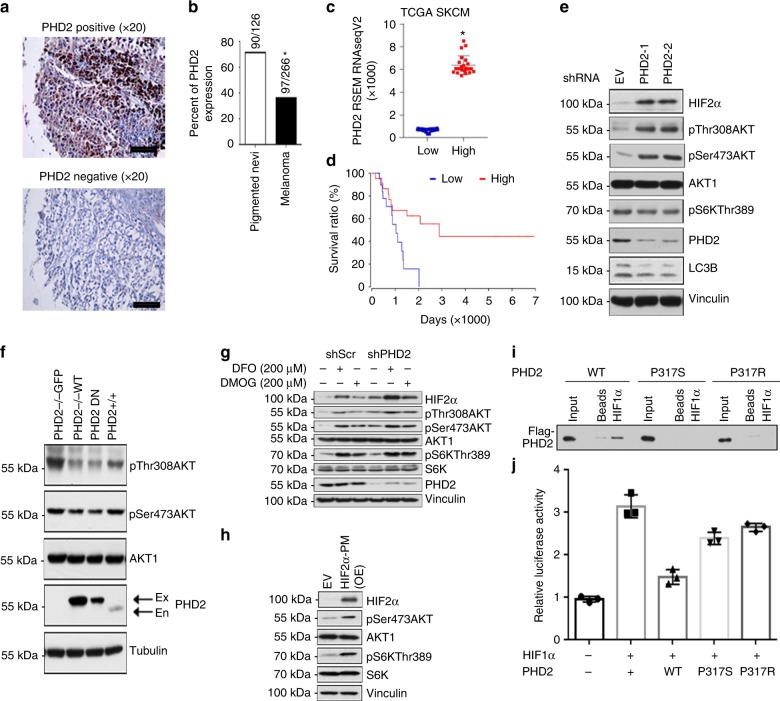
PHD2 expression and function in human melanocytes and melanomas. **a** IHC staining of PHD2 in 126 nevus and 266 melanoma specimens. A melanoma progression tissue microarray (TMA) was stained with the anti-PHD2 antibody and PHD2 staining positive and negative cases were calculated. Representative PHD2 positive (upper panel) and negative cases (lower panel) are shown. Bars indicate 100 µm. **b** Percentage of cases with PHD2 expression. There was significantly less percentage of melanoma cases with PHD2 expression than that of nevi; *p < 0.01. **c**. TCGA SKCM patients were sorted into PHD2-high and PHD2-low groups according to *PHD2* mRNA expression levels (top 5% versus bottom 5%). **d** *PHD2* expression and survival curve. The Kaplan–Meier survival curves showed that the *PHD2*-high group have a significantly improved patient survival than *PHD2*-low group (log rank *p* value = 0.00643). **e** *PHD2* knockdown induces Akt phosphorylation. Immortalized human melanocytes (hTERT/p53DD/Cdc24R24C-BRAFV600E) were transfected with two independent PHD2 shRNAs. Cells transfected with shGFP were included as a negative control. Western blots were performed using the indicated antibodies. **f** Reintroduction of *PHD2* in *PHD2*^−/−^ MEFs reduces Akt phosphorylation. PHD2^−/−^ MEFs (left lane) were transfected with wild-type PHD2 (lane adjacent to the left lane). PHD2-positive MEFs which express endogenous PHD2 were used as a control (right lane). **g** DFO or DMOG treatment. Immortalized human melanocytes were transfected with control vectors or EGLN knockdown vectors. These cells were than treated with DFO or DMOG. **h** Overexpressed HIF-2α in immortalized human melanocytes. **i** The melanoma-derived PHD2-P317 mutation inhibits PHD2 binding to HIF-1α. Biotin-labeled HIF1α-ODD peptides were incubated with WCLs derived from HEK293T cells transfected with the indicated PHD2 constructs (beads as negative control). Binding of PHD2 to HIF1α-ODD is abolished with the P317 mutation. **j** The PHD2-P317 mutation inhibits PHD2 and HIF-1α interaction. Unlike WT-PHD2, P317S-PHD2 is largely impaired in inhibiting HRE reporter activities detected by the Dual-Luciferase Reporter Assay System. The error bars indicate s.d.

To study the function of PHD2 in melanoma progression, we first determined expression of PHD2 by immunohistochemistry in a melanoma tissue microarray composed of 480 cores of benign nevi and melanomas^22^. PHD2 staining was evaluated in 126 cores of nevi and 266 cores of melanomas (Fig. 1a). We found that PHD2 was expressed in nevi with a higher percentage (90 out of 126; 71.4%) than that in melanomas (97 out of 266; 36.5%) (*p* = 0.0174) (Fig. 1b). To study the potential relationship between *PHD2* expression and patient survival, we first sorted The Cancer Genome Atlas (TCGA) skin cutaneous melanoma (SKCM) patients into *PHD2*-high and *PHD2*-low groups according to *PHD2* messenger RNA (mRNA) expression levels. As shown in Fig. 1c, the *PHD2*-high group represents an average RNA-Seq by Expectation Maximization (RSEM) level of 6385 (*n* = 22), which is significantly higher than *PHD2*-low group with an average RSEM level of 663.6 (*n* = 22). Next, we analyzed the difference in survival between these two groups using OncoLnc (http://www.oncolnc.org/). The Kaplan–Meier survival curves showed that the *PHD2*-high group have a significantly improved patient survival than *PHD2*-low group (Log rank *p* value = 0.00643) (Fig. 1d).

Although molecular mechanisms underlying the transformation of nevus to melanoma have not been completely elucidated, the Akt–mTOR pathway has been implicated^23^. Thus, we tested whether decreased expression of PHD2 can activate the Akt pathway in human melanocytes. To this end, we knocked down *PHD2* in immortalized human melanocytes (IHPM-VE cells) using two different short hairpin RNAs (shRNAs), and found that knockdown of *PHD2* led to stabilization of HIF-2α with elevated levels of Akt phosphorylation at both p-T308 and p-S473 as well as decreased expression of LC3B, an autophagy marker (Fig. 1e). To confirm a causal relationship between decreased PHD2 expression and elevated Akt activity, *PHD2* was reintroduced into *PHD2* knockout murine embryonic fibroblast (MEF) cells. Expression of *PHD2* led to a significant reduction in the phosphorylation of Akt, particularly at T308 (Fig. 1f), which is indicative of reduced Akt activity. To study the interaction between *PHD2* knockdown and hypoxia, we treated the immortalized human melanocytes after *PHD2* knockdown with desferrioxamine (DFO) or dimethyloxalylglycine (DMOG, a cell-permeable prolyl-4-hydroxylase inhibitor) (Fig. 1g). Notably, DFO or DMOG treatment induced a further modest increase of Akt activity. When we overexpressed *HIF-2α* in the human melanocytes, moderately increased Akt phosphorylation was also observed (Fig. 1h). Similar results were observed after *Braf*^*V600E*^; *Phd2*^*−/−*^ melanoma cells cultured under 1% oxygen (Supplementary Fig. 1a) or expression of a non-degradable *HIF-1α* (Supplementary Fig. 1b). Interestingly, PHD2 expression was also regulated by hypoxia. However, the effect of hypoxia on PHD2 expression appeared to be cell line specific as two melanoma cell lines showed increased expression while the other two did not show significant change (Supplementary Fig. 2a–d).

We analyzed the TCGA SKCM data then discovered a novel *PHD2* mutation, P317S, in a melanoma patient. Interestingly, patients carrying a P317R mutation developed erythrocytosis, but its mechanistic connection to melanomagenesis is unknown^24^. Because the P317R mutation reduced HIF hydroxylase activity and the ability of PHD2 to interact with HIF, we examined whether the PHD2-P317S mutation was also deficient in its interaction with and subsequent hydroxylation of HIF. To this end, we carried out peptide binding assays by incubating biotin-labeled HIF1α-ODD peptides with transiently transfected *PHD2* wild type (WT) or mutants (P317S and P317R). Notably, the WT, but not P317R or P317S PHD2, was capable of interacting with HIF1α-ODD (Fig. 1i), indicating that the Proline-317 residue plays a pivotal role in mediating the interaction of PHD2 with the well-characterized HIF1α-ODD domain. In keeping with this notion, we conducted reporter assays. The results of these assays showed that compared with WT-PHD2, the PHD2 mutant P317S could not efficiently suppress the transcriptional activity of HIF-1α in cells (Fig. 1j), indicating that this mutation causes a loss-of-function phenotype.

### Homozygous *Phd2* deletion cooperates with BRaf^V600E^ to induce melanoma in vivo

To further define the role of *Phd2* in melanomagenesis in vivo, we generated *Tyr::CreER; Phd2*^*lox/lox*^ mice. The topical administration of 4-hydroxytamoxifen (4-OHT) to either fur-bearing or glabrous skin did not lead to the appearance of any pigmented lesions over 18 months (Supplementary Fig. 3a). In contrast, the topical administration of 4-OHT to either fur-bearing or glabrous skin led to the development of *nevi* with the appearance of pigmented patches (melanocytic nevi) in the *Tyr::CreER*; *BRaf*^CA^ compound transgenic mouse strain (Supplementary Fig. 3b). Nevertheless, none of nevi progressed to primary melanoma after 1.5 years, which is consistent with a previous report^25^. Thus, our data indicate that *Phd2* deletion alone is not sufficient to induce melanocytic hyperplasia.

**Fig. 2:**
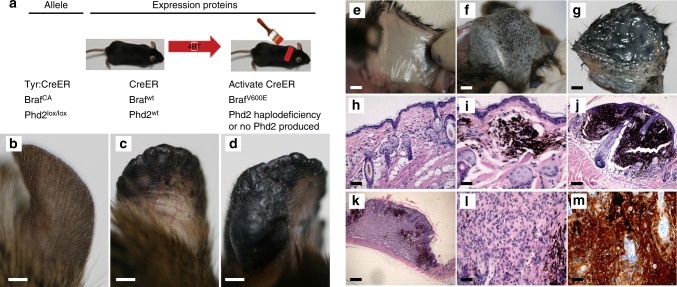
*Phd2* loss cooperates with *BRaf*^V600E^ to induce malignant melanoma in mice. **a** Treatment scheme. Mice carrying various conditional alleles of *BRaf* (*BRaf*^CA^) and/or *Phd2* (*Phd2*^lox/lox^) were crossed to the *Tyr::CreER* mice with melanocyte-specific expression of a hormone-dependent form of *Cre* recombinase (*CreERT2*). 4-OHT-dependent activation of the *CreER* recombinase leads to a melanocyte-specific conversion of *BRaf*^CA^→*BRaf*^V600E^ and the conversion of the *Phd2*^lox^ alleles to *Phd2*^−/−^ alleles. **b**–**g** Melanoma progression in mice. *Tyr::CreER;BRaf*^*CA*^;*Phd2*^*lox/lox*^ mice were treated topically with 4-OHT on the ear (**b**–**d**) and flank (**e**–**g**). 4-OHT-treated mice were killed at 40, 80 and 120 days after the induction. The pigmented lesions at flank and ear was photographed at days 40 (**b**, **e**), 80 (**c**, **f**), and 120 (**d**, **g**) following the 4-OHT induction. **h**–**m** Histology of melanoma in mice. Mouse tissues were processed and histology was examined using hematoxylin and eosin stain. Increased pigmented cells in the dermis were present at day 40 (**h**) and pigmented cells formed small tumor nodules in the dermis at day 80 (**i**). At day 120, melanoma cells formed bigger tumor nodules (**j**, **k**). Cytologically, these tumor cells were focally pigmented and had enlarged nuclei, a morphology that is similar to human melanoma (**l**). Tumor cells were stained positive for the S100 antibody (**m**). Bars in **b**–**g** indicate 5 mm. Bars in **h**–**j** indicate 200 μm. Bar in **k** indicates 400 μm. Bars in **l**, **m** indicate 50 μm

Because activation of the Akt–mTOR oncogenic signaling is required for BRAF^V600E^-induced melanomagenesis^26^, we further studied whether the cooperation of *PHD2* deletion and *BRAF*^*V600E*^ is sufficient to trigger melanoma formation. To this end, we generated *Tyr::CreER; Braf*^*CA*^; *Phd2*^*lox/lox*^ compound mice. After performing PCR to confirm the genotype of these engineered mice (Supplementary Fig. 4a), we administered 4-OHT topically on the ear, flank and tail of *Tyr::CreER; BRaf*^*CA*^*; Phd2*^*lox/lox*^ mice at 6 weeks of age (Fig. 2a). After 40 days, there was little gross pigmentation in the areas (ear and flank) where 4-OHT was administered (Fig. 2b, e). However, the histologic analysis showed an increase in the number of solitary melanocytes in the dermis (Fig. 2h). Flat pigmented lesions began to appear 80 days after the administration of 4-OHT (Fig. 2c, f) which continued to grow and formed tumor nodules after another 40 days (Fig. 2d, g). Histologically, there were increasing numbers of pigmented tumor cells in the dermis, forming small dermal aggregates (Fig. 2i) to expansile nodules (Fig. 2j). After 4–5 months, fully developed melanoma occupied the entire dermis and subcutis (Fig. 2k). Mouse melanoma cells showed enlarged nuclei and contained nucleoli (Fig. 2l) which exhibited features consistent with those of human melanoma cells.

Pagetoid proliferation of melanocytes is a histological hallmark for human melanoma^27^. Examination of the epidermis from induced mice revealed pagetoid proliferation of melanocytes (Supplementary Fig. 4b). Some of mouse melanoma cells were heavily pigmented and tumor cells were diffusely positive for S100 (Fig. 2m), confirming their melanocytic lineage. The deletion of *Phd2* in mouse melanoma tissues was also confirmed by PCR (Supplementary Fig. 4c). Notably, 100% of the *Tyr::CreER; BRaf*^*CA*^*; Phd2*^*lox/lox*^ mice developed melanoma upon the administration of 4-OHT, whereas none of mice treated with vehicle control developed melanoma (Supplementary Fig. 3c).

**Fig. 3:**
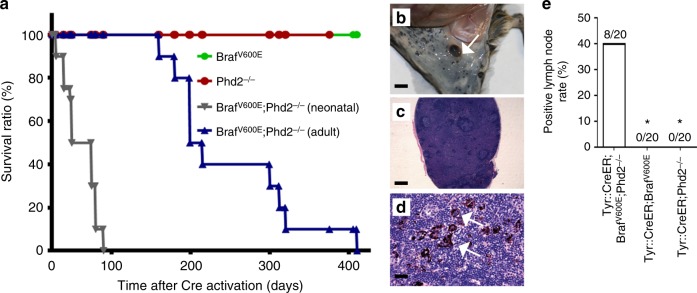
Survival and lymph node metastasis in *Tyr::CreER*;*BRaf*^V600E^;*Phd2*^−/−^ mice. **a** Kaplan–Meier survival analysis of *Tyr::CreER*;*BRaf*^V600E^ (*n* = 20), *Tyr::CreER*;*Phd2*^−/−^ (*n* = 20), *Tyr::CreER*;*BRaf*^V600E^;*Phd2*^−/−^ postnatal mice (*n* = 20) and *Tyr::CreER*;*BRaf*^V600E^;*Phd2*^−/−^ adult mice (6 weeks, *n* = 20) after 4-OHT induction. Log rank tests of survival plots of the data indicated a statistically significant difference between the following survival curves: *Tyr::CreER*; *BRaf*^*V600E*^; versus *Tyr::CreER*;*BRaf*^*V600E*^;*Phd2*^−/−^ mice (*p* < 0.0001), and *Tyr::CreER*;*BRaf*^*V600E*^;*Phd2*^−/−^ adult versus *Tyr::CreER*;*BRaf*^*V600E*^;*Phd2*^−/−^ postnatal mice (*p* < 0.0001). **b**–**d** Lymph node metastasis in *Tyr::CreER*;*BRaf*^*V600E*^;*Phd2*^−/−^ mice. A positive lymph node was present near the area with melanoma induction (arrow points to the lymph node, **b**). The lymph node was enlarged with pigmented cells (**c**) and high-power view showed melanoma cells in the lymph node (arrows point to the tumor cells, **d**). **e** Percentage of mice that developed lymph node metastasis. Bar in **c** indicates 200 μm. Bar in **d** indicates 50 μm. Bar in **b** indicates 3 mm. *p < 0.05 compared to Tyr::CreER;Braf^V600E^;Phd2^−/−^ group using one-way ANOVA with pairwise comparisons

The life span of *Tyr::CreER; BRaf*^*CA*^*; Phd2*^*lox/lox*^ mice was significantly shorter than that of *Tyr::CreER; BRaf*^*CA*^ or *Tyr::CreER; Phd2*^*lox/lox*^ mice (Fig. 3a). Interestingly, when 4-OHT was administered in *Tyr::CreER; BRaf*^*CA*^*; Phd2*^*lox/lox*^ mice during the neonatal period (the first 3 days after the birth), the initiation of melanoma occurred earlier than that in adult mice. All neonatal mice required killing within 95 days following the administration of 4-OHT (Fig. 3a).

Upon necropsy, we found that grossly positive lymph nodes were common in *Tyr::CreER; BRaf*^*V600E*^; *Phd2*^*−/−*^ mice (Fig. 3b). The histologic examination further confirmed the presence of metastatic melanoma cells in lymph nodes (Fig. 3c, d). Notably, 40% of adult *Tyr::CreER; BRaf*^*V600E*^; *Phd2*^*−/−*^ mice developed lymph node metastasis, whereas 60% of mice developed lymph node metastasis when 4-OHT was administered during the neonatal period (Fig. 3e). Nevertheless, we did not observe any distant organ metastasis (lung, liver, spleen and heart) in these mice. Therefore, our data demonstrate that the cooperation of homozygous *Phd2* deletion with oncogenic *BRAF* leads to the development of primary cutaneous melanoma with lymph node metastatic capacity in mice.

### Haplo-deficient Phd2 induces melanoma with longer latency in vivo

**Fig. 4:**
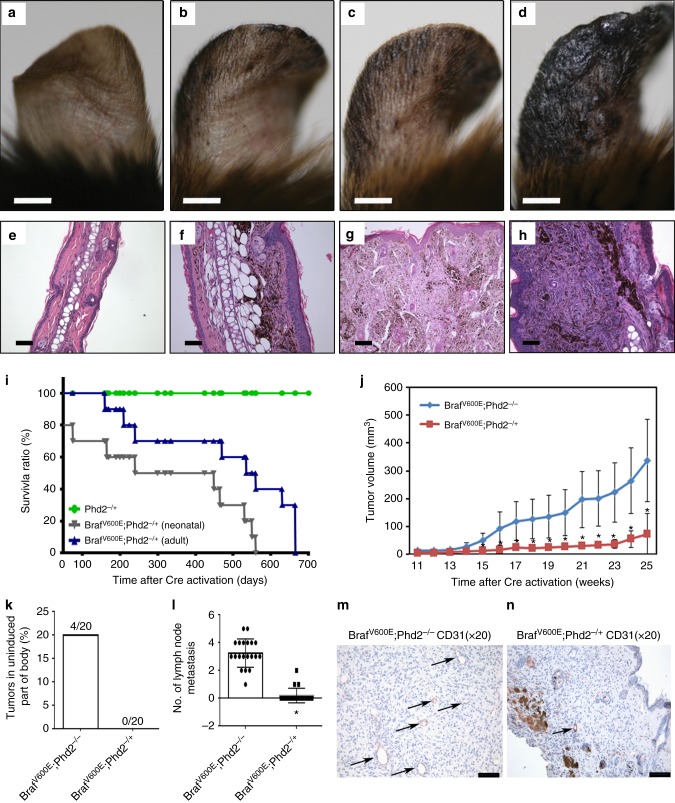
Haplo-deficient *Phd2* is sufficient to cooperate with *BRaf*^V600E^ to induce melanoma in mice. **a**–**h** Haplo-deficient *Phd2* is sufficient to cooperate with BRAF^V600E^ to induce melanoma in mice. The 6-week old adult *Tyr::CreER*; *BRaf*^*CA*^; *Phd2*^lox/+^ mice were treated topically on the ear, flank and tail with 4-OHT. The presence of pigmented lesions was assessed at days 45 (**a**), 90 (**b**), 135 (**c**) and 180 (**d**) following 4-OHT administration. 4-OHT-treated mice were killed at 45, 90 and 180 days after the induction. The pigmented lesions were photographed at day 45 (**e**), 90 (**f**) and 180 (**g**, **h**) following the 4-OHT induction. **i** Kaplan–Meier survival analysis of *Tyr::CreER*; *BRaf*^V600E^; *Phd2*^-/+^ neonatal mice (*n* = 20) and *Tyr::CreER*; *BRaf*^V600E^; *Phd2*^-/+^ (*n* = 20) adult mice (6 weeks to 1 year old). **j** Comparison of tumor volume in mice with homozygous deletion of *Phd2* or haplo-deficient *Phd2* mice. Tumor volume in *Tyr::CreER*; *BRaf*^V600E^; *Phd2*^-/+^ (*n* = 20) mice was significantly smaller than that of *Tyr::CreER*; *BRaf*^V600E^; *Phd2*^−/−^ mice (*p* = 0.00171). **k** Melanoma shown on uninduced part of body (%) in homozygous deletion or haplo-deficient *Phd2* mice. Melanoma present at uninduced body part was quantified (*n* = 20 in each group). **l** Average number of positive lymph nodes in homozygous deletion or haplo-deficient *Phd2* mice (*n* = 20 in each group). **m**, **n** CD31 immunohistochemical stain in in homozygous deletion or haplo-deficient *Phd2* mice (*n* = 5 in each group). Bars in **a**–**d** indicate 1 mm. Bars in **e**–**h**, **m**, **n** indicate 100 μm. Arrows in **m**, **n** point to CD31+ blood vessels. *p < 0.05 (*t*-test) compared to Brafv600E;Phd2^−/−^ group in **j** and **l**; error bars indicate s.d.

It has been shown that *Phd2* haplo-deficient (*Phd2*^*+/−*^) mice have an enhanced angiogenic response to ischemia and that the combined loss of *Phd2* in stromal and cancer cells sensitize tumors to chemotherapy^28^. Hence, we attempted to address whether *Phd2* haplo-deficiency in melanocytes is still capable of cooperating with oncogenic BRAF to induce melanoma in our experimental setting. After genotyping *Tyr::CreER; BRaf*^*CA*^*; Phd2*^*lox/+*^ mice (Supplementary Fig. 4a), we topically treated 6-week-old mice on the ear, flank and tail with 4-OHT. The presence of pigmented lesions was assessed at 45 (Fig. 4a), 90 (Fig. 4b), 135 (Fig. 4c) and 180 (Fig. 4d) days following the 4-OHT administration. Pigmented tumor did arise but the latency of appearance of pigmented lesions was relatively longer than that in *Tyr::CreER*; *BRaf*^V600E^; *Phd2*^−/−^ mice. Histologically, there were increasing numbers of individual pigmented tumor cells in the dermis, forming small dermal aggregates (Fig. 4e) to expansile nodules (Fig. 4f, g). After 6 months, fully developed melanoma occupied the entire dermis (Fig. 4h).

When 4-OHT was administered during the neonatal period (the first 3 days after the birth), melanoma was induced in *Tyr::CreER; BRaf*^*CA*^*; Phd2*^*lox/+*^ mice earlier than that in adult mice as all mice required killing within 570 days following the administration of 4-OHT (Fig. 4i). After 4-OHT administration, 100% of the *Tyr::CreER*; *BRaf*^CA^; *Phd2*^lox/+^ mice eventually developed melanoma. The Kaplan–Meier survival analysis showed that *Tyr::CreER*; *BRaf*^CA^; *Phd2*^lox/+^ mice survived significantly longer than those *Tyr::CreER*; *BRaf*^CA^; *Phd2*^lox/lox^ mice after the 4-OHT administration (Fig. 4i). Moreover, tumor volumes in *Tyr::CreER*; *BRaf*^CA^; *Phd2*^lox/+^ mice were significantly smaller than that of *Tyr::CreER*; *BRaf*^CA^; *Phd2*^lox/lox^ mice after the administration of 4-OHT (Fig. 4j). 20% of *Tyr::CreER*; *BRaf*^V600E^; *Phd2*^*−/−*^ mice developed tumor outside the area treated with 4-OHT, while none of the *Tyr::CreER*; *BRaf*^V600E^; *Phd2*^-/+^ mice developed tumor outside the area treated with 4-OHT (Fig. 4k). An average of 3.5 positive lymph nodes were identified in *Phd2*^*−/−*^ mice, while few positive lymph nodes were seen in PHD2^*−*/+^ mice (Fig. 4l). There were more CD31-positive blood vessels in *Phd2*^*−/−*^ tumors (Fig. 4m) than that in the *Phd2*^*−*^^/+^ melanomas (Fig. 4n). Taken together, our data indicate that while the effect of haplo-deficient *Phd2* is weaker than that of biallelic deletion of *Phd2*, it is still sufficient to cooperate with mutant BRAF to induce melanoma.

### Phd2 deletion increases HIF target expression and glucose uptake

**Fig. 5:**
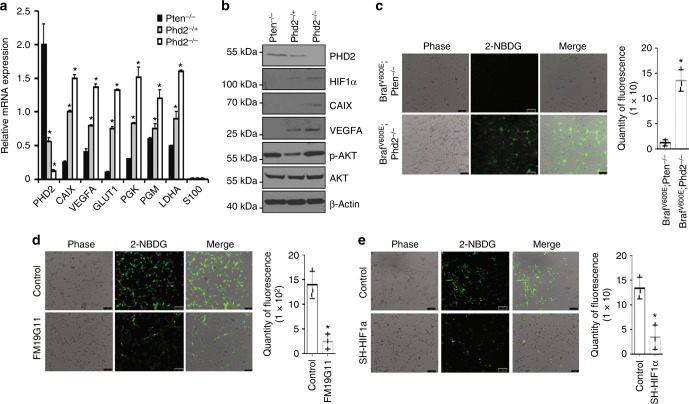
*Phd2* deletion induced HIF target and glucose uptake changes. **a** *Phd2* regulated gene expression in mouse melanomas. Quantitative RT-PCR assay for *Phd2*, *CAIX*, *VEGFA*, *GLUT1*, *PGK*, *PGM* and *LDHA* mRNA expression in *Tyr::CreER*; *BRaf*^V600E^; *Phd2*^−/−^ mice melanoma tissues (*n* = 3 replicate experiments; *p < 0.01 compared with *Tyr::CreER; BRaf*^*V600E*;^ *Phd2*^−/+^ or *Tyr::CreER; BRaf*^*V600E*;^ *Pten*^−/−^ mouse melanoma tissues). β-Actin is used as loading control. **b** *Phd2* regulated protein expression in mouse melanomas. Expression of Phd2, HIF-1α, VEGFA, pAkt and Akt protein was determined by western blot analysis in *Tyr::CreER*; *BRaf*^V600E^; *Phd2*^*−/−*^, *Tyr::CreER*; *BRaf*^V600E^; *Phd2*^*-*/+^ and *Tyr::CreER*; *BRaf*^V600E^; *Pten*^*−/−*^ mouse melanoma tissues (*n* = 3 replicate experiments). β-Actin was used as a loading control. **c** Uptake of a fluorescent deoxyglucose analog (2-NBDG) is increased in *Phd2*^−/−^ mouse melanoma cells. The 2-NBDG uptake assay was performed using *BRaf*^V600E^; *Phd*2^−/−^ or *BRaf*^V600E^; *Pten*^−/−^ mouse melanoma cells (left panel) (*n* = 3 replicate experiments). Quantity of 2-NBDG uptake is assessed in these mice (right panel) (*n* = 3 replicate experiments, *p < 0.05). **d** HIF inhibition reverses *Phd2* deletion-induced glucose uptake increase. 2-NBDG uptake is assessed in FM19G11-treated *BRaf*^*V600E*^; *Phd2*^*−/−*^ melanoma cells (*n* = 3 replicate experiments, *p < 0.05 compared with control group). **e** Knockdown of *HIF-1α* reverses *Phd2* deletion-induced glucose uptake increase. 2-NBDG uptake is assessed in *BRaf*^*V600E*^; *Phd2*^*−/−*^ melanoma with *HIF-1α* knockdown (*n* = 3 replicate experiments, *p < 0.05 compared with control group). One way ANOVA or *t*-test was used for statistical analysis and error bars indicate s.d.

To elucidate mechanisms underlying melanomagenesis in *Tyr::CreER; Braf*^V600E^; *Phd2*^*−/−*^ mice, we first studied gene expression in haplo-deficient and homozygous *Phd2*-depleted mouse tumor tissues in comparison to that in *Tyr::CreER*; *BRaf*^V600E^; *Pten*^−/−^ mice by quantitative reverse transcription PCR (RT-PCR). We found that melanomas with haplo-deficient *Phd2* expressed significantly less *Phd2* than that in *Phd2* wild-type mice. The tumors with homozygous deletion of *Phd2* expressed even less *Phd2* and the residual Phd2 expression was probably due to normal stromal tissue residing within the melanomas (Fig. 5a). Phd2 target genes were significantly elevated in melanomas with haplo-deficient Phd2 and more so in tumors with homozygous deletion of *Phd2* (Fig. 5a). Western blot analysis of melanoma tissues showed increased HIF-1α, carbonic anhydrase IX (CAIX) and vascular endothelial growth factor A (VEGFA) in melanomas with haplo-deficient *Phd2* and even more pronounced increase in homozygous deletion of *Phd2* compared with tumor tissues from *Tyr::CreER; Braf*^V600E^; *Pten*^−/−^ mice (Fig. 5b). Melanoma with haplo-deficient *Phd2* deletion showed less pAkt level than that of melanoma with *Pten* deletion (Fig. 5b). We then established a melanoma cell line (*Braf*^*V600E*^; *Phd2*^−/−^) from the tumors that developed in *Tyr::CreER; Braf*^V600E^; *Phd2*^*−/−*^ mice (Supplementary Fig. 5a, b). We used 2-NBDG, a fluorescent tracer, to monitor glucose uptake into living cells. The results showed that *Braf*^*V600E*^; *Phd2*^*−/−*^ melanoma cells had significantly more glucose uptake than that of *Braf*^*V600E*^; *Pten*^−/−^ melanoma cells (Fig. 5c). Increased glucose uptake in *Braf*^V600E^; *Phd2*^−/−^ melanoma cells was significantly inhibited by FM19G11 treatment, a HIF inhibitor (Fig. 5d) or *HIF-1α* knockdown (Fig. 5e), which is accompanied by concomitant decreased Glut1 expression (Supplementary Fig. 6).

### Phd2 deletion leads to Akt–mTOR pathway activation

**Fig. 6:**
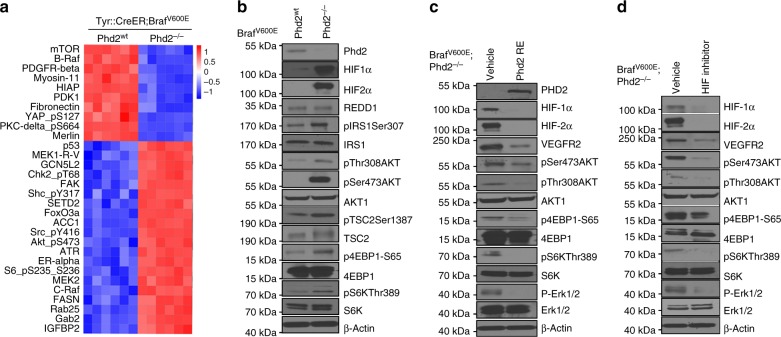
*Phd2* deletion leads to activation of the Akt–mTOR pathway. **a** RPPA analysis of tumors with homozygous deletion of *Phd2*. Tumor tissues from *Tyr::CreER*; *BRaf*^V600E^; *Phd2*^−/−^ or *Tyr::CreER*; *BRaf*^V600E^ mice were processed and analyzed by RPPA assays. The analyses identified proteins that were significantly changed in mouse melanomas compared to *nevi*. **b** Activation of Akt–mTOR pathway after *phd2* deletion. Tumor tissues were processed and western blots showed stabilization of HIF-1α and HIF-2α proteins after *Phd2* depletion. Increased phosphorylation of Akt, 4EBP1 and S6K was observed in tumors from *Tyr::CreER*; *BRaf*^*V600E*^; *Phd2*^−/−^ compared with those of *Tyr::CreER*; *BRaf*^*V600E*^ mice. **c** Re-expression of *Phd2* inhibits the Akt–mTOR pathway. A *BRaf*^*V600E*^; *Phd2*^*−/−*^ mouse melanoma cell line was established from melanomas in *Tyr::CreER*; *BRaf*^*CA*^; *Phd2*^lox/lox^ mice. *Phd2* was ectopically reintroduced in these tumor cells. Western blot analysis showed that degradation of HIF-1α and HIF-2α proteins with decreased expression of VEGFR2 decreased phosphorylation of Akt, 4EBP1 and S6K. **d** Pharmacological inhibition (FM19G11) of HIF pathway in *BRaf*^*V600E*^; *Phd2*^*−/−*^ melanoma cells. A similar but more pronounced inhibition of the Akt–mTOR pathway was observed using the HIF inhibitor. β-Actin was used as a loading control. Results are representative of three independent experiments

We isolated proteins from melanocytic nevi derived from *Tyr::CreER; Braf*^V600E^ mice and melanomas derived from *Tyr::CreER; Braf*^V600E^; *Phd2*^*−/−*^ mice and profiled expression of 291 signaling transduction proteins using the reverse phase protein arrays (RPPA). The analyses of RPPA data identified 20 proteins that were significantly up-regulated in mouse melanomas compared to nevi (Fig. 6a). Of note, 4 of them were phospho-AKT^S473^, phospho-S6^S235/236^, Phospho-Shc^Y317^ and phospho-Src^Y416^. Our western blot showed elevated expression of phospho-AKT^S473^, phospho-S6K^T389^ and phospho-4EBP1^S65^ in mouse melanomas compared to nevi (Fig. 6b). We performed immunohistochemical stains and showed that the melanoma cells were positive for phospho-4EBP1 and phospho-S6K (Supplementary Fig. 7a, b).

To confirm a causal relationship between the loss of *Phd2* and the elevation in Akt pathway activity, we re-expressed *Phd2* in the *Braf*^*V600E*^*; Phd2*^*−/−*^ melanoma cells and found that re-expression of *Phd2* in these cells induced a significant decrease in HIF-1α and HIF-2α protein expression and a decrease in the phosphorylation of Akt and 4EBP1 compared to those of control (Fig. 6c). Notably, there was no significant change in the phosphorylation of extracellular signal-regulated kinase-1/2 (ERK1/2). To further study whether HIF pathway is involved in the regulation of Akt pathway by *Phd2* deletion, we treated *Braf*^*V600E*^*; Phd2*^*−/−*^ melanoma cells with FM19G11. The treatment led to a decrease in phosphorylation of Akt, 4EBP1 and S6K compared to those of cells treated with the vehicle control (Fig. 6d). In addition, we performed silencing *HIF-1α* and *HIF-2α* in the *Braf*^*V600E*^*; Phd2*^*−/−*^ melanoma cells and showed a decreased activation of Akt pathway (Supplementary Fig. 8a, b). We also treated the *Braf*^*V600E*^*; Phd2*^*−/−*^ melanoma cells under 1% oxygen and demonstrated that hypoxia further stabilized HIF-1α and HIF-2α expression with increased pAkt and pS6K levels (Supplementary Fig. 1a). Furthermore, we transfected the *Braf*^*V600E*^*; Phd2*^*−/−*^ melanoma cells with a non-degradable HIF-1α that also resulted in elevated pAkt and pS6K levels (Supplementary Fig 1b). Taken together, our data support that *Phd2* deletion activates the Akt pathway in melanomas, in part, via HIFs.

### Inhibition of mTOR pathway suppresses melanoma growth in vivo

**Fig. 7:**
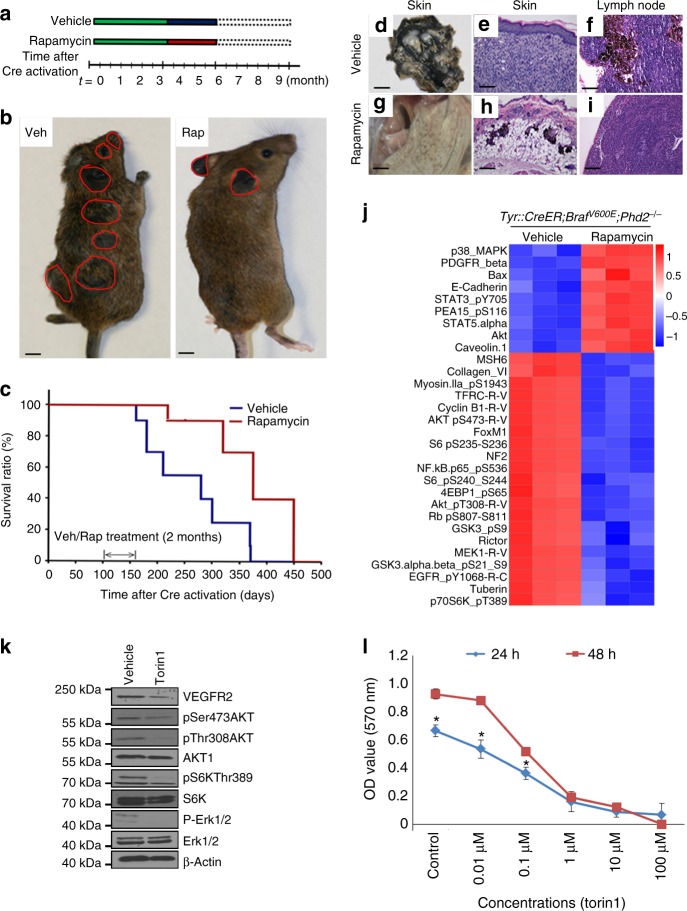
Inhibition of mTOR pathway suppresses the growth of melanoma in vivo. **a** Scheme of rapamycin treatment. **b** Representative images of *Tyr::CreER*; *BRaf*^*V600E*^; *Phd2*^−/−^ mouse treated with the vehicle control (left panel) or rapamycin (right panel). Rapamycin treatment lasted for 8 weeks. Tumor masses are highlighted by red circles. **c** Kaplan–Meier survival curves of mice treated with vehicle control (*n* = 10) or rapamycin (*n* = 10) and *p* < 0.05. **d**–**i** Representative images of skin and lymph nodes in mice treated with the vehicle control or rapamycin. After killing the mice, underside of skin was exposed and photographed (**d**, **g**). The entire dermis was occupied by tumor cells (**e**) and lymph node was positive for melanoma in the vehicle-treated mice (**f**). On the contrary, there were significantly fewer tumor cells in the dermis (**h**) with negative lymph node (**i**) in the rapamycin-treated mice. **j** Inhibition of the Akt–mTOR pathway resulted in tumor growth suppression. Tumor tissues with or without rapamycin treatment were processed and analyzed using RPPA assays. Proteins with most significant changes were used to generate the heatmap. Notably, phospho-Rb^S807/811^, phospho-AKT^S473^, Cyclin B1, TFRC, phospho-S6^S235/236^, phospho-EGFR^Y1068^, MEK1 and phospho-AKT^T308^ proteins were significantly reduced in mice treated with rapamycin. **k** Torin1 inhibits Akt–mTOR pathway. *BRaf*^*V600E*^; *Phd2*^−/−^ melanoma cells were treated with torin1 for 48 h. Western blot analysis was performed. Results are representative of three independent experiments. **l** Torin1 inhibits *BRaf*^*V600E*^; *Phd2*^−/−^ melanoma cell growth. The melanoma cells were treated with different concentrations of torin1 (Control, 0.01, 0.1, 1, 10 and 100 μM) and different lengths of time (24 or 48 h). Results are summary of three independent experiments. Bars in **b** indicate 6 mm. Bars in **d**, **g** indicate 3 mm. Bars in **e**, **f**, **h**, **i** indicate 100 µm. *p < 0.05 compared to control (*t*-test); error bars indicate s.d.

Because the AKT–mTOR–S6 signaling axis was activated during melanomagenesis in *Tyr::CreER; Braf*^V600E^*; Phd2*^*−/−*^ mice, we reasoned that rapamycin, which targets the mTORC1 kinase complex, may inhibit melanomagenesis induced in these engineered mice. Melanoma was induced in these mice and when tumors become palpable, we treated these mice with the vehicle control or rapamycin (1.5 mg/kg/day) for 8 weeks and then these mice were followed (Fig. 7a). Tumor growth was significantly inhibited by rapamycin treatment (Fig. 7b). In addition, administration of rapamycin prolonged the life span of *Tyr::CreER; Braf*^V600E^*; Phd2*^*−/−*^ mice (median survival time = 375 days) compared to those mice treated with the vehicle control (median survival time = 275 days) (*p* = 0.0018) (Fig. 7c). Vehicle-treated mice developed large pigmented tumors (Fig. 7d). In some mice, the treatment with rapamycin induced the complete regression of mouse melanoma (Fig. 7g). Histologic analysis confirmed that significantly smaller cluster of tumor cells and aggregates of melanophages were localized to the dermis in *Tyr::CreER; Braf*^V600E^*; Phd2*^*−/−*^ mice treated with rapamycin (Fig. 7h) compared to bulky tumors in the control group (Fig. 7e). In contrast to the mice treated with vehicle (Fig. 7f), lymph node metastasis was not seen in treated mice (Fig. 7i). In order to investigate the signaling pathways that are altered in *Tyr::CreER; Braf*^V600E^*; Phd2*^*−/−*^ mice after treatment with rapamycin, we conducted RPPA assays using tissues collected before and after treatment. The analysis of RPPA data identified 21 proteins that were significantly downregulated by rapamycin. Many of the downregulated proteins are regulated by the Akt–mTOR–S6 signaling pathway, such as: phospho-Rb^S807/811^, phospho-AKT^S473^, Cyclin B1, TFRC, phospho-S6^S235/236^, phospho-EGFR^Y1068^, MEK1 and phospho-AKT^T308^ (Fig. 7j). Torin1 inhibitor is a potent inhibitor of mTORC1/2^29^. It inhibited pAkt and pS6K levels in *Braf*^*V600E*^*; Phd2*^*−/−*^ melanoma cells (Fig. 7k), and inhibited the proliferation of *Braf*^*V600E*^*; Phd2*^*−/−*^ melanoma cells in a dose-dependent manner (Fig. 7l). The proliferation of *Braf*^*V600E*^*; Phd2*^*−/−*^ melanoma cells was also significantly slowed down after silencing *HIF-1α* or *HIF-2α* (Supplementary Fig. 9a). However, silencing *HIF-1α* or *HIF-2α* added little additional inhibitory effect to torin1 treatment on the proliferation of *Braf*^*V600E*^*; Phd2*^*−/−*^ melanoma cells (Supplementary Fig. 9b). Taken together, our data further support that the activation of the Akt–mTOR–S6 signaling axis that occurs as a result of *Phd*2 deletion is crucial for melanoma cell proliferation.

## Discussion

Our study is the first to demonstrate that PHD2 plays a crucial tumor suppressor role in the initiation of melanoma. Although the effect of haplo-deficiency of *Phd2* in melanocytes is relatively weaker than that exhibited when biallelic deletion of *Phd2* occurs, it is still sufficient to cooperate with oncogenic *BRAF* to induce melanoma. This observation suggests that the intact PHD2 signaling pathway is vital for its tumor suppressor role. Both mouse and human melanoma data support that the tumor suppression function of PHD2 is mediated through the AKT–mTOR pathway.

The tumor suppressor function of PHD2 is supported by several clinical and pathological studies which showed that the decrease in expression of PHD2 is associated with worse clinical outcomes^30–32^. In this study, we also demonstrated a decreased expression of PHD2 during progression of nevi to melanomas, and analysis of the TCGA SKCM data revealed that lower expression of *PHD2* is associated with worse clinical outcome. Our RPPA data reveal that *Phd2* deletion in melanocytes leads to activation of the AKT–mTOR signaling pathway. Re-expression of *Phd2* in *Phd2*^*−/−*^ melanoma cells suppresses the Akt–mTOR pathway, further supporting the involvement of this pathway. The degree of Akt pathway activation in melanoma cells with *Phd2* deletion is less than that in melanoma cells with *Pten* deletion, which may be one of the reasons that melanoma has a longer latency in *Tyr::CreER; Braf*^V600E^*; Phd2*^−/−^ mice than that in the *Tyr::CreER; Braf*^V600E^*; Pten*^−/−^ mice. Activation of Akt pathway is critical for *Phd2*^*−/−*^ melanoma cell proliferation since suppression of this pathway using torin1 or rapamycin significantly inhibits *Braf*^V600E^*; Phd2*^*−/−*^ melanoma cell growth in vitro and mouse melanomas in *Tyr::CreER; Braf*^V600E^*; Phd2*^*−/−*^ mice and prolongs their lifespans, respectively. The activation of AKT pathway was also observed in immortalized human melanocytes after *PHD2* knockdown and the phenotype can be rescued by reintroduction of PHD2. These results support that the tumor suppressor function of PHD2 is mediated at least in part through the AKT–mTOR pathway.

*Phd2* deletion results in stabilization of HIF-1α and HIF-2α in *Braf*^*V600E*^*; Phd2*^*−/−*^ melanoma cells. Our analysis of TCGA SKCM data discovers a novel *PHD2-*P317S mutation in melanoma and this mutation also resulted in deficiency of PHD2 interaction with HIF-1α in human melanocytes. HIF inhibitor or *HIF-1α* knockdown suppresses AKT–mTOR pathway in *Braf*^*V600E*^*; Phd2*^−/−^ melanoma cells, supporting the involvement of HIF pathway activation in mediating the effect of *Phd2* deletion, at least in melanoma setting. The AKT–mTOR and HIF pathways are closely intertwined^33^. AKT–mTOR regulates HIF-1α synthesis^34^ while HIF-1α has been shown to regulate Akt–mTOR signaling in cancer^35^. Another recent study showed that the conditional deletion of HIF-1α or HIF-2α in melanocytes failed to inhibit melanomagenesis in *Tyr::CreER; Braf*^V600E^; *Pten*^−/−^ mice. However, HIF-1α or HIF-2α independently contributed to melanoma metastasis^36^. These results might not be unexpected since our data demonstrated that *Phd2* deletion has a weaker effect on Akt pathway activation than that of *Pten* deletion in melanocytes. Deletion of HIF-1α or HIF-2α may be insufficient to overcome the strong tumor promoting effect of PTEN deletion. Our results support that HIF pathway is important for the tumor suppressor function of PHD2. Nevertheless, our recent study also showed that PHD2 can directly proline hydroxylate AKT on two major proline residues that can inactivate AKT^37^ and deleting PHD2 can activate AKT in a VHL-dependent manner. Thus, it is likely that PHD2 depletion can activate AKT in both HIF-independent and HIF-dependent pathways, in different cellular or tissue contexts.

In summary, our studies provide evidence that PHD2 functions as a tumor suppressor through suppression of the AKT–mTOR oncogenic pathway in melanoma. Increased HIF activity is associated with its enhancement of anaerobic metabolism, neoangiogenesis and aggressive growth of malignancies^38,39^. Since PHIs are been tested in multiple phase III clinical trials in anemic patients with chronic renal failure (ClinicalTrials.gov), the long-term effects of PHIs should be carefully observed in these treated patients for their possible effect on tumor growth. This is particularly important clinically since uremic patients are considered to be immunocompromised. That being said, we cannot exclude the possibility that there may be contributions to the tumor suppressor role of PHD2 that are independent of its catalytic activity^17^. If this were the case, it might mitigate concerns over potential pro-tumorigenic effects of PHIs. Future studies are warranted to address this critical issue in both melanoma and other human cancer settings.

## Methods

### Cell culture

WM115A, 1205Lu, 451Lu and WM3918 cells were kind gifts from Meenhard Herlyn (The Wistar Institute, Philadelphia, PA). Melanoma cells were cultured under 37 °C humidified atmosphere containing 5% CO_2_ and grown in MCDB153/L15 medium (4:1, v/v) supplemented with 2% fetal bovine serum, insulin (5 units/mL), CaCl_2_ (2 mmol/L), 100 units/mL penicillin and 100 mg/mL streptomycin^40^. We used VenorTM GeM Mycoplasma Dection Kit, PCR-based (Catalog Number: MP0025) to rule out mycoplasma infection.

### Melanoma tissue microarray and IHC staining

The melanocytic tumor progression tissue microarray (TMA) includes 130 cores from 35 nevi, 200 cores from 60 primary melanoma and 150 cores from 75 metastatic lesions^41^. Histological sections of the tissue array slide TMA sections were processed using an automated immunohistochemistry (IHC) platform (Benchmark, Ventana, Roche, Illkirch, France) and sections were stained with a polyclonal PHD2 antibody in a 1:100 dilution (Novus Biologicals; catalog number NB100-137).

### PHD2 expression and patient survival

To study the possible relationship between *PHD2* gene expression and survival, we first sorted the TCGA SKCM patient into *PHD2*-high and *PHD2*-low groups according to PHD2 mRNA expression levels (top 5% versus bottom 5%). Next we analyzed the difference in survival between these two groups using OncoLnc* (www.oncolnc.com).

### Plasmids and reagent

pLenti-EglN1 mammalian expression plasmid, HIF-responsive element (HRE)-containing plasmid and lentiviral shRNA constructs targeting human EglN1, EglN2 and EglN3 were obtained from Dr. William G. Kaelin, Jr (Dana Farber Cancer Institute, Boston). Site-directed mutagenesis was performed to generate various EglN1 mutants using the QuikChange XL Site-Directed Mutagenesis Kit (Stratagene, City) according to the manufacturer’s instructions. SH-HIF1a and SHI-HIF2a lenti plasmids were gifts from Dr. M. Celeste Simon (Perelman School of Medicine, University of Pennsylvania). pCDNA3-HIF1a was constructed by Dr. Frank Lee. Deoxy-2-[(7-nitro-2,1,3-benzoxadiazol-4-yl)amino]-d-glucose (catalog number 72987) was purchased from Sigma and Torin1 (catalog number S2827) from Selleckchem.

### Transfection and infection

MEFs (ATCC), 293FT (ATCC) and 293T (ATCC) cells were cultured in Dulbecco's modified Eagle's medium (DMEM) supplemented with 10% fetal bovine serum (FBS) and 1% penicillin/streptomycin (Pen/Strep). Immortal human melanocytes (hTERT/p53^DD^/Cdk4^R24C^/BRAF^V600E^ (ATCC) (IHPM-VE) were cultured in glutamine containing Ham’s F12 media supplemented with 7% FBS, 0.1 mM IBMX, 50 ng/mL TPA, 1 µM Na_3_VO_4_ and 1 µM dbcAMP^42^. The 293FT packaging cell lines were used for the lentiviral production. Lentiviral infection was carried out as follows: post transfection with Lipofectamine 2000 (Invitrogen), viruses were collected after 48 and 72 h^43^. After passing through 0.45 μM filters, appropriate amounts of viruses were used to infect target cells in the presence of 8 μg/mL polybrene. Following the lentiviral infection, cells were maintained in the presence of puromycin at 2 μg/mL for at least 72 h to eliminate any uninfected cells. All cells were maintained at 37 °C in 5% CO_2_.

### Immunocytochemistry and immunoblotting

Expression of S100, p4EBP1, pS6K and CD31 was detected by immunocytochemistry using the commercially available anti-S100 (4C4.9, catalog number GTX24066, GeneTex Inc, Irvine, CA, USA), anti-p4EBP1 (catalog number 2855, Cell Signaling Technology), anti-pS6K (catalog number PA5-38307, Thermo Fisher Scientific) and anti-CD31(catalog number AF3628-SP, R&D). Cells or sections were stained with antibodies in a 1:100 dilution. The EBC buffer (50 mM Tris pH 8.0, 120 mM NaCl, 0.5% NP40, 0.1 mM EDTA and 10% Glycerol) supplemented with the complete protease inhibitor cocktail (Roche Applied Biosciences) was used to extract the whole cell lysates.

Anti-VEGF-A (AB1876-I, Millipore Sigma), anti-VEGFR2 (Ab-1059, catalog number 21531-1, Signalway Antibody), anti-CAIX (catalog number 11071-1-AP, Proteintech), anti-REDD1 (catalog number ABC245, EMD Millipore), anti-alpha Tubulin (ab4074, Abcam), anti-IRS1 (ab5603, Abcam), anti-pIRS1Ser307 (catalog number 2381s), anti-TSC2 (catalog number 3612), anti-pTSC2Ser1387 (catalog number 5584s), anti-p-4EBP1-S65 (catalog number 9451S), anti-4EBP1 (catalog number 9452S), anti-S6K (catalog number 9202), anti-p-Erk1/2 (catalog number 4370S), anti-Erk1/2 (catalog number 4695S), anti-pT308-Akt (catalog number 13038S), anti-pS473-Akt (catalog number 4060S), anti-Akt (catalog number 4685), anti-pT389-S6K (catalog number 9205S), anti-LC3 (catalog number 2775) and anti-EglN1 (catalog number 4835) antibodies were purchased from Cell Signaling Technology. Anti-HIF2α (H1alpha67, catalog number NB100-105), anti-HIF2α (catalog number NB100-132 Lot13), anti-EglN2 (catalog number NB 100-310SS) and anti-EglN3 antibodies (catalog number NB 100-139SS) were purchased from Novus Biological. Anti-FLAG (A2220), anti-β-actin (A1978) and anti-vinculin (V9131) were obtained from Sigma (Sigma-Aldrich, Cream Ridge, NJ, USA). Antibodies are diluted 1:1000 for western blot. Full immunoblots are shown in Supplementary Fig. 10.

### Peptide binding assays

HIF1α peptides were a gift from Dr. Qing Zhang (UNC Chapel Hill, City, USA) and were synthesized at The W.M. Keck Biotechnology Resource Center at Yale University. They were synthesized at 25 μM scale, with the N-terminus biotinylated and the C-terminal end remaining free. The sequences are listed as: 556-575 DLDLEMLAPYIPMDDDFQLR. For peptide binding assays, 1 μg of peptides was incubated at 4 °C for 4 h with 1000 μg of whole cell extracts recovered from the addition of EBC buffer to 293T cells transfected with FLAG-EglN1 WT or FLAG-EglN1 P317S or P317R. Then, 10 µl of streptavidin agarose beads was added and the mixture was incubated for another 1 h. Then, the beads were washed 5 times with NETN buffer (100 mM NaCl, 20 mM Tris-HCl, pH 8.0, 0.5 mM EDTA and 0.5% (v/v) Nonidet P-40). The bound proteins were eluted after being boiled in sodium dodecyl sulfate (SDS) loading buffer, resolved by SDS–polyacrylamide gel electrophoresis and detected by immunoblot analysis.

### Luciferase reporter assays

The 293T cells were transiently transfected with HRE plasmids and/or HIF-1α combined with or without different EglN1 mutants. After 36 h of transfection, cells were harvested and luciferase activities were measured by a Dual-Luciferase Reporter Assay System (Promega) according to the manufacturer’s instructions.

### Transgenic mouse generation and genotyping

All animal procedures were approved by the Institutional Animal Care and Use Committees at the University of Pennsylvania in compliance with Animal Welfare Assurance. The *Phd2*^lox/lox^ mouse line was generated as previously described^44^. Genomic DNA was isolated from mouse tails^45^. *Braf* ^CA^,*Tyr::CreER* and *Phd2*^lox^ was genotyped as follows: *Cre*-mediated conversion of *Braf*^CA^ to *Braf*^V600E^ was determined by standard PCR of tail DNA with primer pair AD (AD FwdA1, 5’-TGAGTATTTTTGTGGCAACTGC-3’; and AD RevB1, 5’-CTCTGCTGGGAAAGCGGC-3’) to produce diagnostic PCR products of 185 bp, 308 bp and 335 bp for BRaf, BRaf CA and the BRaf VE alleles, respectively^46,47^. Reaction conditions were as follows: 92 ^o^C, 2 min, 1 cycle; 92 ^o^C, 30 s, 60 ^o^C, 30 s, 72 ^o^C, 45 s, 34 cycles; 72 ^o^C, 3 min, 1 cycle. For the *Phd2* floxed allele (PCR product = 0.73 kb for wild-type allele, 0.84 kb for floxed allele): PHD2Rec55 primer = 5’-AGG GCT TCT GGC ATT AGT TGA CC-3’ and Pex2-2 3’ primer = 5’-ACA TGT CAC GCA TCT TCC ATC TCC-3’. For the *Phd2* knockout allele (PCR product = 0.56 kb): Pint1 Sal 5’ primer = 5’-AAT GGC TTG GGA CAA CTC-3’ and Pint2-3 3’ primer = 5’-GGA CAA CGT TTG GGA GTT GGT AAG-3’. Reaction conditions were as follows: 94 ^o^C, 2 min, 1 cycle.; 94 ^o^C, 30 s, 58 ^o^C, 30 s, 72 ^o^C, 50 s, 35 cycles; 72 ^o^C, 2 min, 1 cycle. The administration of 4-OHT was conducted as previously described^25^.

### Reverse phase protein array assays (RPPA)

Protein lysates including: (Lysis Buffer: 1% Triton X-100, 50 mM HEPES, pH 7.4, 150 mM NaCl, 1.5 mM MgCl_2_, 1 mM EGTA, 100 mM NaF, 10 mM Na pyrophosphate, 1 mM Na_3_VO_4_, 10% glycerol, containing freshly added protease and phosphatase inhibitors from Roche Applied Science catalog no. 05056489001 and 04906837001, respectively; 4× SDS Sample Buffer including: 40% glycerol, 8% SDS, 0.25 M Tris-HCL, pH 6.8). Before use sample buffer, add 2-mercaptoethanol (at 1 10 of the volume), then added to the lysis were extracted from frozen tumors and profiled by the RPPA platform at the MD Anderson Functional Proteomics Core facility as previously described^48^. A detailed description of the RPPA method, data normalization and the list of 291 antibodies are available at the core facility’s web page. Heatmaps were generated using Cluster and Tree View^48^. The *p* values for differences in protein expression between two treatment groups were determined by BRB-ArrayTools using a random variance model.

### Establishment of mouse melanoma cell lines

Mouse melanoma tissue was washed with DMEM medium once and then minced in 5 mL of DMEM medium. Then, 500 µL of collagen (20 mg/mL) and 200 µL hyaluronidase (20 mg/mL) (catalog no. 070M7033, Sigma) were added and the tissue was digested at 37 ^o^C for 30 min and subject to vortexing once every minute for 10 min. The medium was then filtered through a 40 μm nylon cell strainer and then spun down at 1500 RPM for 7 min. The cell pellet was then cultured in 10% FBS DMEM supplemented with Pen/Strep for 24 h and then in 5% FBS DMEM supplemented with Pen/Strep^49^. We sorted out the CD271-positive melanoma cells using the FACS Aria flow cytometer. The FACS analysis of sorted cells was performed using FloJo™ software.

### Quantitative real-time PCR assay

Total RNA was isolated using RNeasy Kit (Qiagen) followed by complementary DNA (cDNA) synthesis using SuperScript First-Strand Synthesis System (Invitrogen). Quantitative PCR was performed using iQ SYBR green supermix (Bio-Rad Laboratories, Hercules, CA) with specific primers listed as follows. cDNA corresponding to 1  μg RNA was added to the iQsyber green supermix and analyzed with icycler (Bio-Rad Laboratories) according to the manufacturer’s instructions. The thermal profiles were 95 °C for 30 s and 56 °C for 30 s. Melting curve analysis was done for each PCR reaction to confirm the specificity of amplification. At the end of each phase, florescence was measured and used for quantitative purpose^9^. The primers sequences are used as follows: *PHD2* forward primer AGGCAACGGAACAGGCTATG, reverse primer CGCATCTTCCATCTCCATTTG; *CAIX* forward primer CGTGATTCTCGGCTACAACTGA, reverse primer CAATCGTTCGCCCATTCAA; *VEGFA* forward primer TGTCACCACCATGCCATCAT, reverse primer GACCCAAAGTGCTCCTCGAA; *GLUT1* forward primer CGAGGGACAGCCGATGTG, reverse primer TGCCGACCCTCTTCTTTCAT; *PGK* forward primer GGAAGCGGGTCGTGATGA, reverse primer GCCTTGATCCTTTGGTTGTTTG; *PGM* forward primer GGTGTTCTGGGAAAGCAGCAGTTTGA, reverse primer TCGTTTGCTGAGAACTGCTTCCCCAC; *LDHA* forward primer AGGACTTGGCGGATGAGCTT, reverse primer CATCTCGCCCTTGAGTTT GTCT; *β-actin* forward primer GCTGACAGGATGCAGAAGGAG, reverse primer TCAAAGAAAGGGTGTAAAACGC.

### 2-NBDG uptake assay

Cells were plated at 5 × 10^4^/well in 6-well plates and used at subconfluence after 24 h of preincubation Experiments were performed within 48 h^50^. For experiments, all culture medium were removed from each well and replaced with 2 mL of culture medium with or without fluorescent 2-NBDG. Plates were incubated at 37 °C with 5% CO_2_ for 20 min before doing confocal microscopy.

### Rapamycin treatment in vivo

Rapamycin (catalog number NC9362949, LC Laboratories) was dissolved in ethanol at a concentration of 20 mg/mL, filter sterilized and then resuspended in vehicle (0.25% PEG, 0.25% Tween-80) at a concentration of 1 mg/mL. Rapamycin at 1.5 mg/kg was injected^51,52^ intraperitoneally daily for 8 weeks. The vehicle was used as the control.

### Cell proliferation assay

Cell proliferation assay was performed using the Cell Proliferation Kit I (catalog number 11465007001, Roche) according to the manufacturer’s instructions^53^, The half-maximal inhibitory concentration (IC_50_) values were calculated from dose–response curves using Graphpad Prism 5. *Braf*^*V600E*^*phd2*^*−/−*^ cells or *Braf*^*V600E*^*phd2*^*−/−*^ with HIF knockdown cells were treated with torin1 (0, 0.01, 0.1, 1, 10 and 100 µM).

### Statistical analysis

All quantitative data were presented as the mean ± SD and at least three independent experiments were performed. Data were analyzed for normality using the Shapiro–Wilk normality test. Nonparametric data were analyzed with Kruskal–Wallis one-way analysis of variance, followed by Dunn’s multiple comparison test. Data with a normal distribution were analyzed by Student’s test. Statistical significance was determined if two-sided *p* < 0.05.

## Electronic supplementary material

Supplementary Information

## Data Availability

The TCGA data referenced during the study are available in a public repository from the OncoLnc website (http://www.oncolnc.org/). All the other data supporting the findings of this study are available within the article and its supplementary information files and from the corresponding author upon reasonable request.
