# Author Correction: Loss of *Phd2* cooperates with *BRAF*^*V600E*^ to drive melanomagenesis

**DOI:** 10.1038/s41467-019-09195-w

**Published:** 2019-03-11

**Authors:** Shujing Liu, Gao Zhang, Jianping Guo, Xiang Chen, Jingce Lei, Kan Ze, Liyun Dong, Xiangpeng Dai, Yang Gao, Daisheng Song, Brett L. Ecker, Ruifeng Yang, Caitlin Feltcher, Kai Peng, Cheng Feng, Hui Chen, Rebecca X. Lee, Heddy Kerestes, Jingwen Niu, Suresh Kumar, Weiting Xu, Jie Zhang, Zhi Wei, James S. Martin, Xiaoming Liu, Gordon Mills, Yiling Lu, Wei Guo, Lunquan Sun, Lin Zhang, Ashani Weeraratna, Meenhard Herlyn, Wenyi Wei, Frank S. Lee, Xiaowei Xu

**Affiliations:** 1 Department of Pathology and Laboratory Medicine, Perelman School of Medicine, University of Pennsylvania, Philadelphia, PA 19104 USA; 2 The Wistar Institute, Philadelphia, PA 19104 USA; 3 Department of Pathology, Beth Israel Deaconess Medical Center, Harvard Medical School, Boston, MA 02115 USA; 4 Xiangya Hospital, Central South University, Changsha, 410008 China; 5 Department of Computer Science, New Jersey Institute of Technology, Newark, NJ 07102 USA; 6 Department of Systems Biology, The University of Texas MD Anderson Cancer Center, Houston, TX 77030 USA; 7 Department of Biology, School of Arts and Sciences, University of Pennsylvania, Philadelphia, PA 19104 USA; 8 Center for Research on Reproduction & Women’s Health, University of Pennsylvania, Philadelphia, PA 19104 USA

Correction to: *Nature Communications* 10.1038/s41467-018-07126-9, published online 21 December 2018

The original version of this Article contained an error in the spelling of the author Brett L. Ecker, which was incorrectly given as Brett Ecker. This has now been corrected in both the PDF and HTML versions of the Article.

