# Supplementary material for: Loss of Phd2 cooperates with BRAFV600E to drive melanomagenesis
Source: Nat Commun. 2018 Dec 21;9:5426. doi: 10.1038/s41467-018-07126-9 (PMC6303344; doi:10.1038/s41467-018-07126-9)
Supplement: Supplementary file 1 — Supplementary Information [file 41467_2018_7126_MOESM1_ESM.pdf]

Liu, et al., Loss of *Phd2* Cooperates with *BRAF*^{V600E} to Drive Melanomagenesis
Supplementary Information

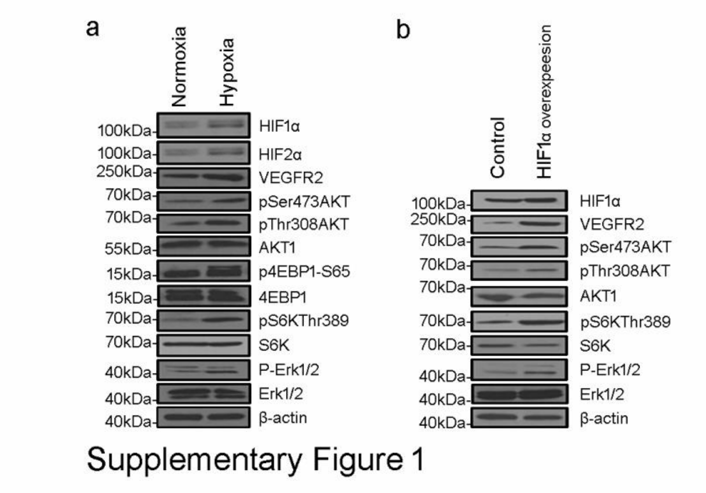

Supplementary Figure 1. Hypoxia regulates Akt-mTOR pathway in *BRaf*^{V600}*E;Phd2*$^{-/-}$ melanoma cells. a.1% oxygen condition further increased HIF stabilization in *BRaf*^{V600}*E; Phd2*$^{-/-}$ melanoma cells and activated Akt-mTOR pathway after the tumor cells were cultured under 1% hypoxia for 24 hours. **b.** Non-degradable *HIF-1α* was expressed in *BRaf*^{V600}*E; Phd2*$^{-/-}$ melanoma cells. β-actin was used as a loading control. Results are representative of 3 independent experiments.

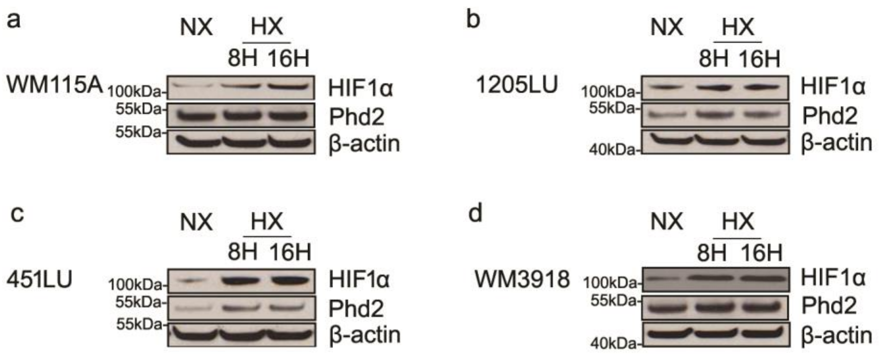

Supplementary Figure 2

Supplementary Figure 2. PHD2 expression under hypoxia in human melanoma cells. a-d. Western Blot assay for PHD2 expression. Human melanoma cells were cultured under hypoxia (1% oxygen) for 8 hours or 16 hours. PHD2 expression in response to hypoxia is cell line dependent. Human WM115A (**a**), 1205LU (**b**), 451LU (**c**) and WM3918 (**d**). *β-actin* was used as loading control. Results are representative of 3 independent experiments.

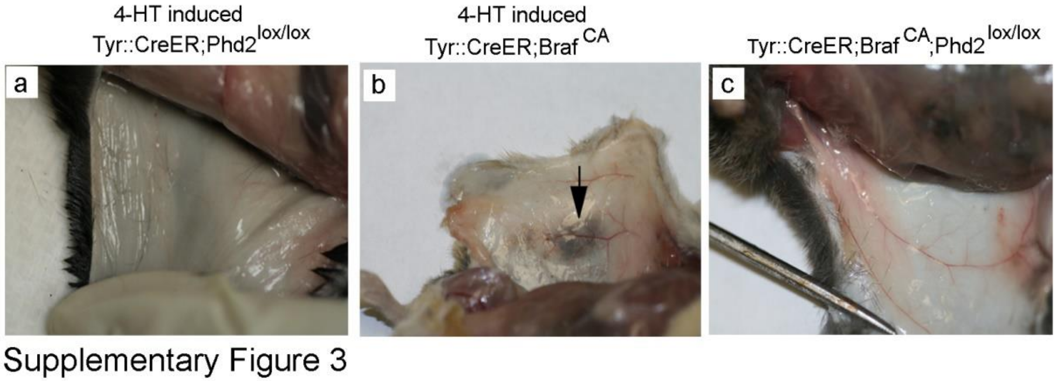

Supplementary Figure 3. Gross skin morphology in different genetically engineered mouse models. Mice were followed for at least 18 months and then sacrificed. The skin was turned upside down and then photographed. 4-OHT induced *Tyr::CreER; Phd2$^{lox/lox}$* **(a),** 4-OHT induced *Tyr::CreER; BRaf^{CA}* **(b),** *Tyr::CreER; BRaf^{CA}; Phd2$^{lox/lox}$* mice without the 4-OHT induction **(c).** Arrow points to the pigmented lesion. Bars indicate 3 mm.

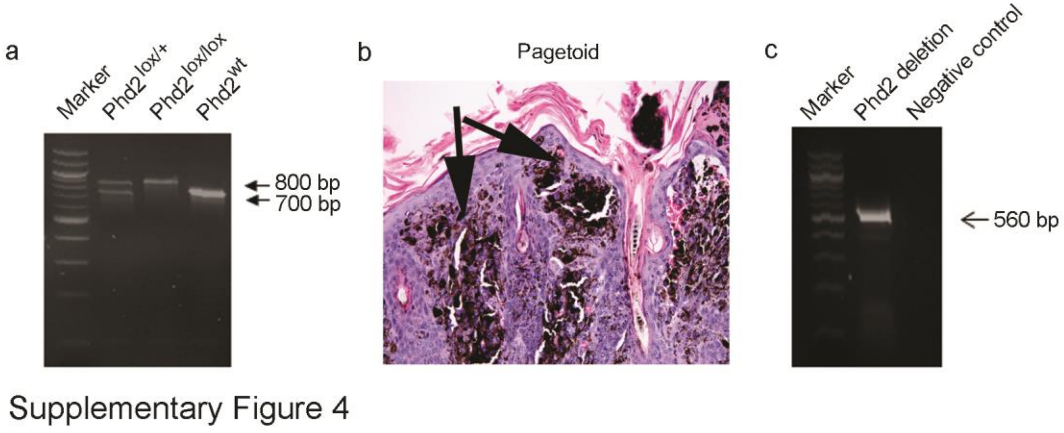

Supplementary Figure 4: **Genotyping of the *phd2* allele. a.** PCR of *phd2* allele in *Phd2*$^{+/+}$, and *Phd2*$^{lox/+}$, and *Phd2*$^{lox/lox}$ mice. **b.** Pagetoid proliferation of melanoma cells in the epidermis derived from the *Tyr::CreER*; *BRaf*^{V600E}; *Phd2*$^{-/-}$ mice. Arrows point to the pagetoid cells. Bar indicates 50 µm. **c.** PCR confirms *Phd2* deletion in melanomas from *Tyr::CreER*;*BRaf*^{V600E};*Phd2*$^{-/-}$ mice.

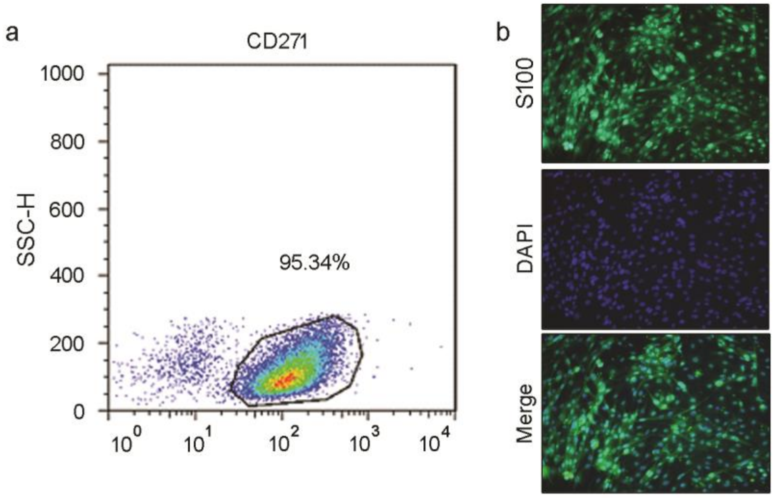

Supplementary Figure 5

Supplementary Figure 5. Generation and characterization of *BRaf*^{V600E}; *Phd2*$^{-/-}$ melanoma cell line. Melanoma cell lines were derived from tumors developed in the *Tyr::CreER*; *BRaf*^{V600E}; *Phd2*$^{-/-}$ mice. **a.** Melanoma was excised from the *Tyr::CreER*; *BRaf*^{V600E}; *Phd2*$^{-/-}$ mice after 4-OHT induction. The tumor sample was dispersed into single cells and CD271+ cells were sorted out using FACS. **b.** The cell line was stained with the anti-S-100 antibody and all the tumor cells were positive for S-100. Bar indicates 50 μm.

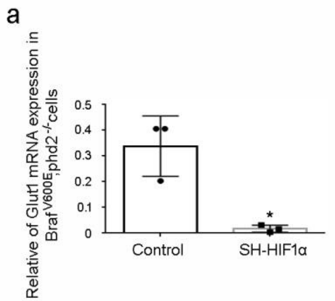

Supplementary Figure 6

Supplementary Figure 6. ***Glut1*** **gene expression is decreased after** ***HIF-1α*** **knockdown in** ***Braf^{V600E},Phd$^{-/-}$*** **melanoma cells.** Quantitative RT-PCR assay for *Glut1* mRNA expression was performed in *Braf^{V600E},Phd$^{-/-}$* melanoma cells with or without *HIF1α* knockdown (n = 3 *replicate* experiments; * indicates $P < 0.01$). *β-actin* was used as control.

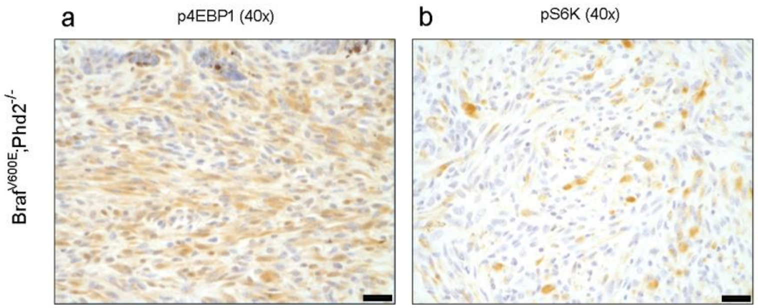

Supplementary Figure 7

Supplementary Figure 7. Immunohistochemistry of *Braf^{V600E},Phd$^{-/-}$* melanoma. Melanomas from the *Tyr::CreER*; *BRaf*^{V600E}; *Phd2*$^{-/-}$ mice were stained with anti-p4EBP1 (**a**) or anti-pS6K antibody (**b**). Bars indicates 20 µm.

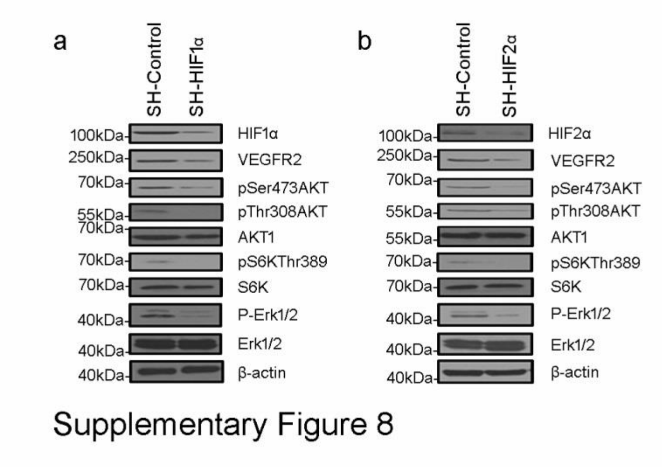

Supplementary Figure 8. Effects of HIF knockdown on Akt-mTOR pathway. Inhibition of the Akt-mTOR pathway was observed in *BRaf*^{V600E}; *Phd2*$^{-/-}$ melanoma cells after these cells were transfected with SH-HIF-1α (**a**) or SH-HIF-2α (**b**). β-actin was used as a loading control. Results are representative of 3 independent experiments.

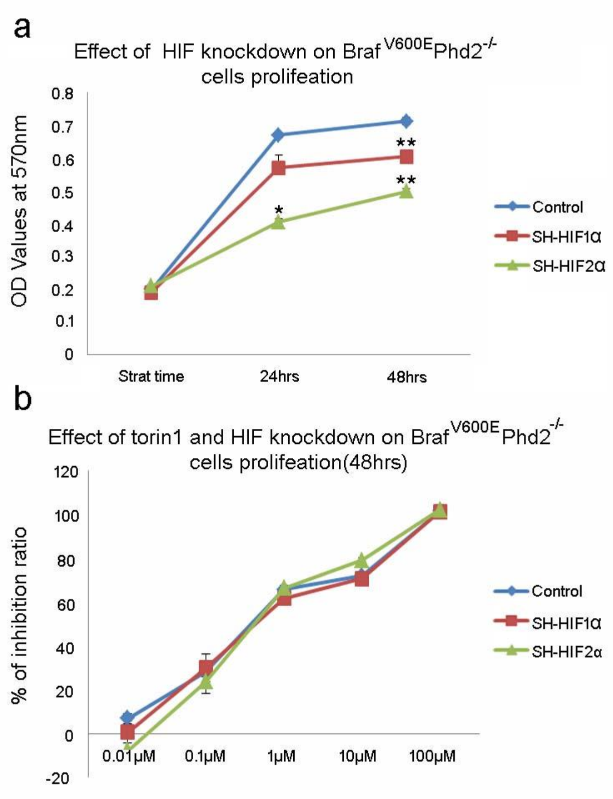

Supplementary Figure 9

Supplementary Figure 9. Effects of HIF knockdown and torin1 on *Braf*^{V600E}*,Phd*$^{-/-}$ melanoma cell proliferation. a. Cell proliferation was measured in *BRaf*^{V600E}; *Phd2*$^{-/-}$ melanomas after *HIF-1α* or *HIF-2α* knockdown. **b**. Cell proliferation was measured in *BRaf*^{V600E}; *Phd2*$^{-/-}$ melanomas after *HIF-1α* or *HIF-2α* knockdown in combination with different concentrations of torin1. Results are from 3 independent experiments.

Supplementary Figure 10

Fig. 1e

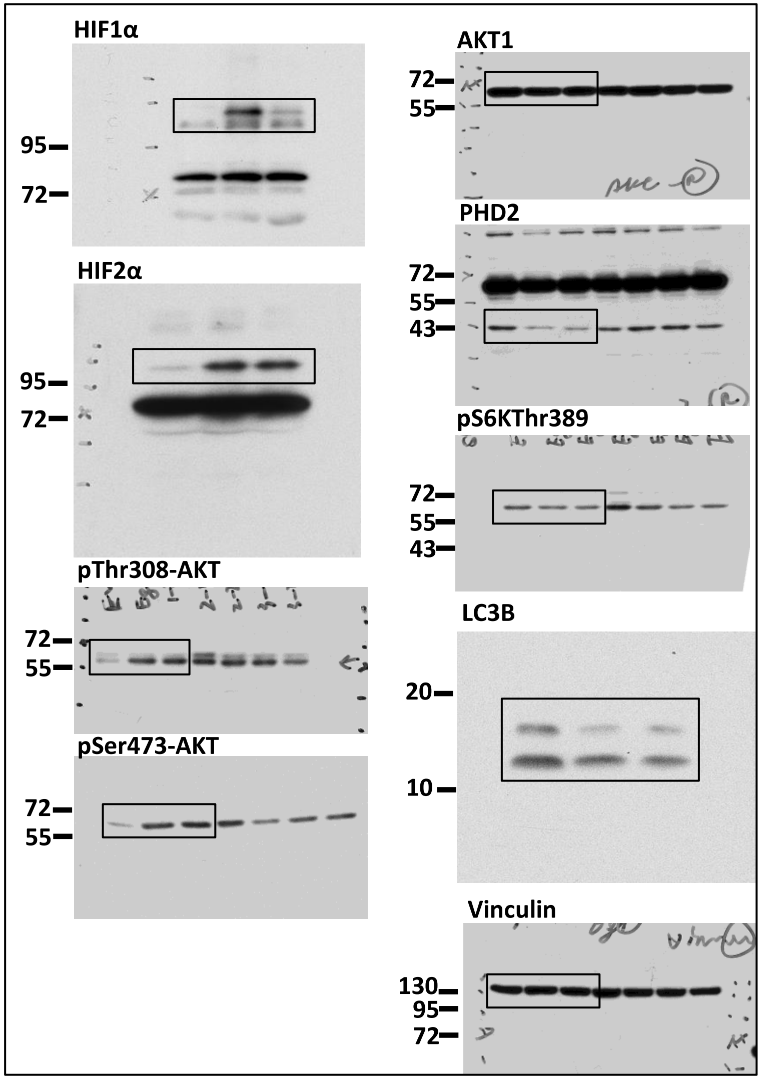

Fig 1f

pT308-Akt

Shorter ep

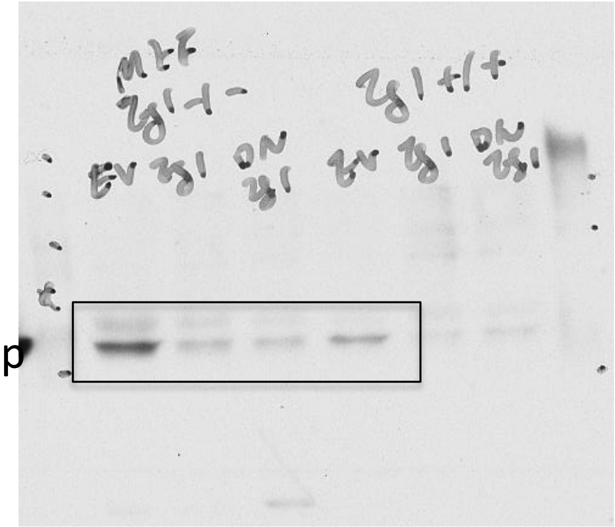

Longer ep

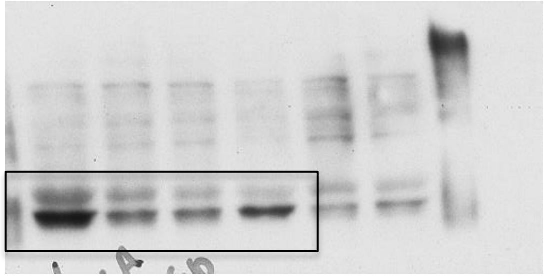

Akt1

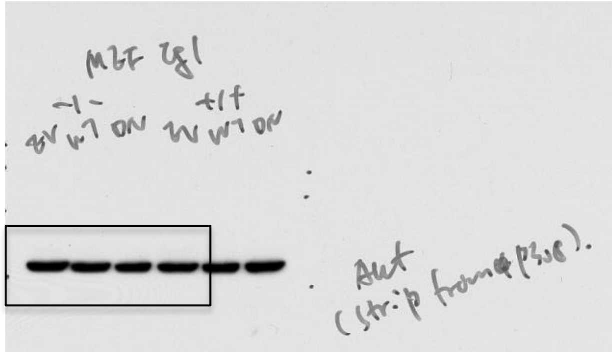

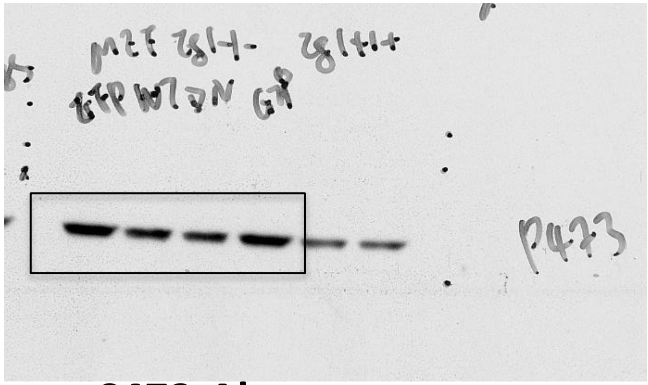

pS473-Akt

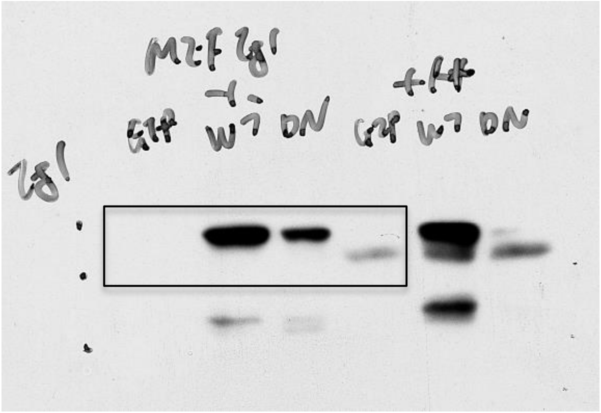

EglN1

Fig. 1g

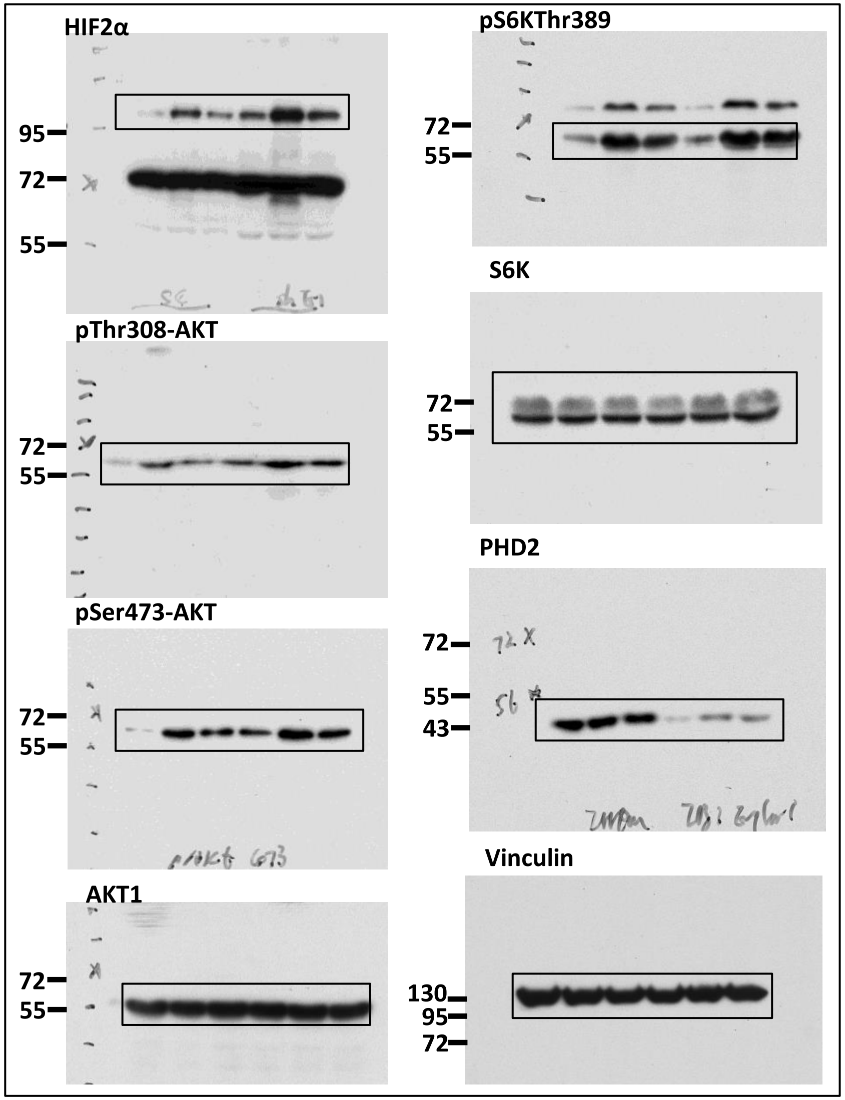
HIF2α
95
72
55
pThr308-AKT
72
55
pSer473-AKT
72
55
AKT1
72
55
pS6KThr389
72
55
S6K
72
55
PHD2
72
55
43
Vinculin
130
95
72

Fig. 1h

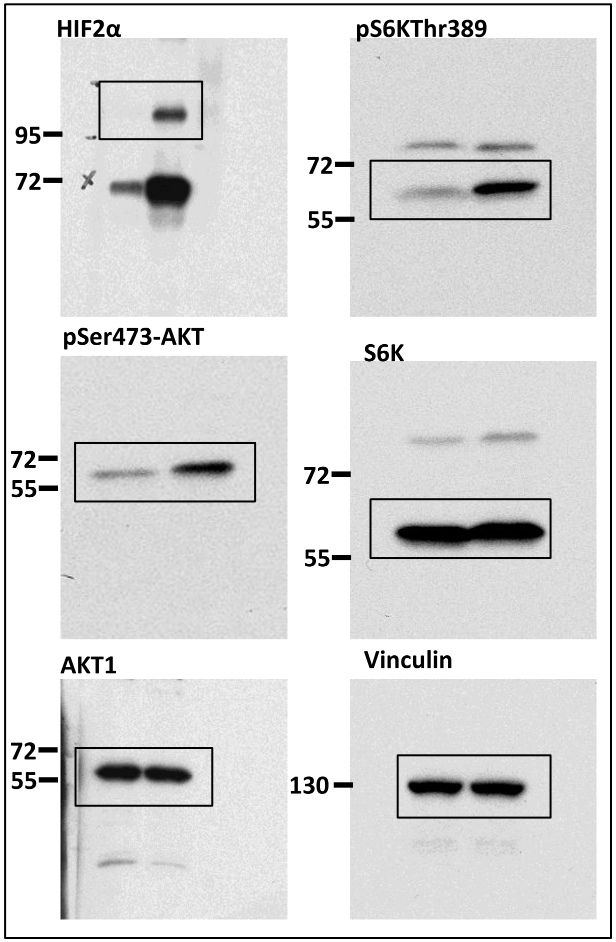

Fig. 1i

Flag-PHD2

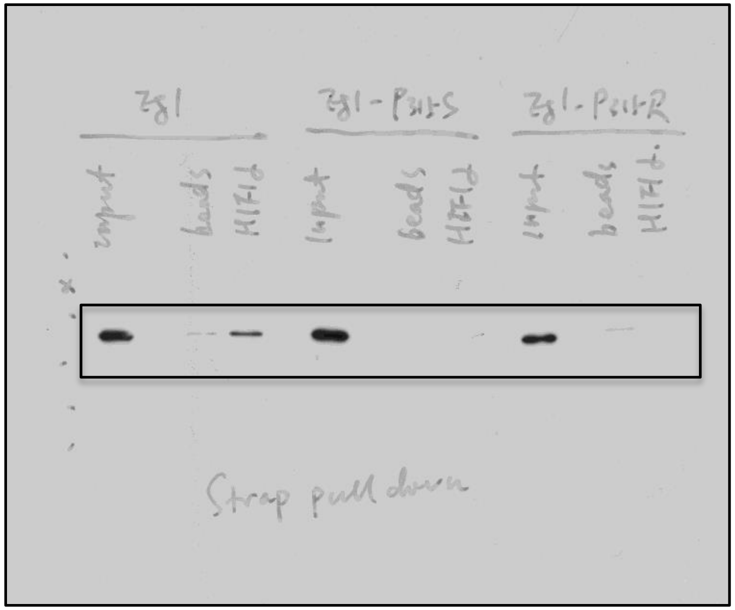

Fig. 5b

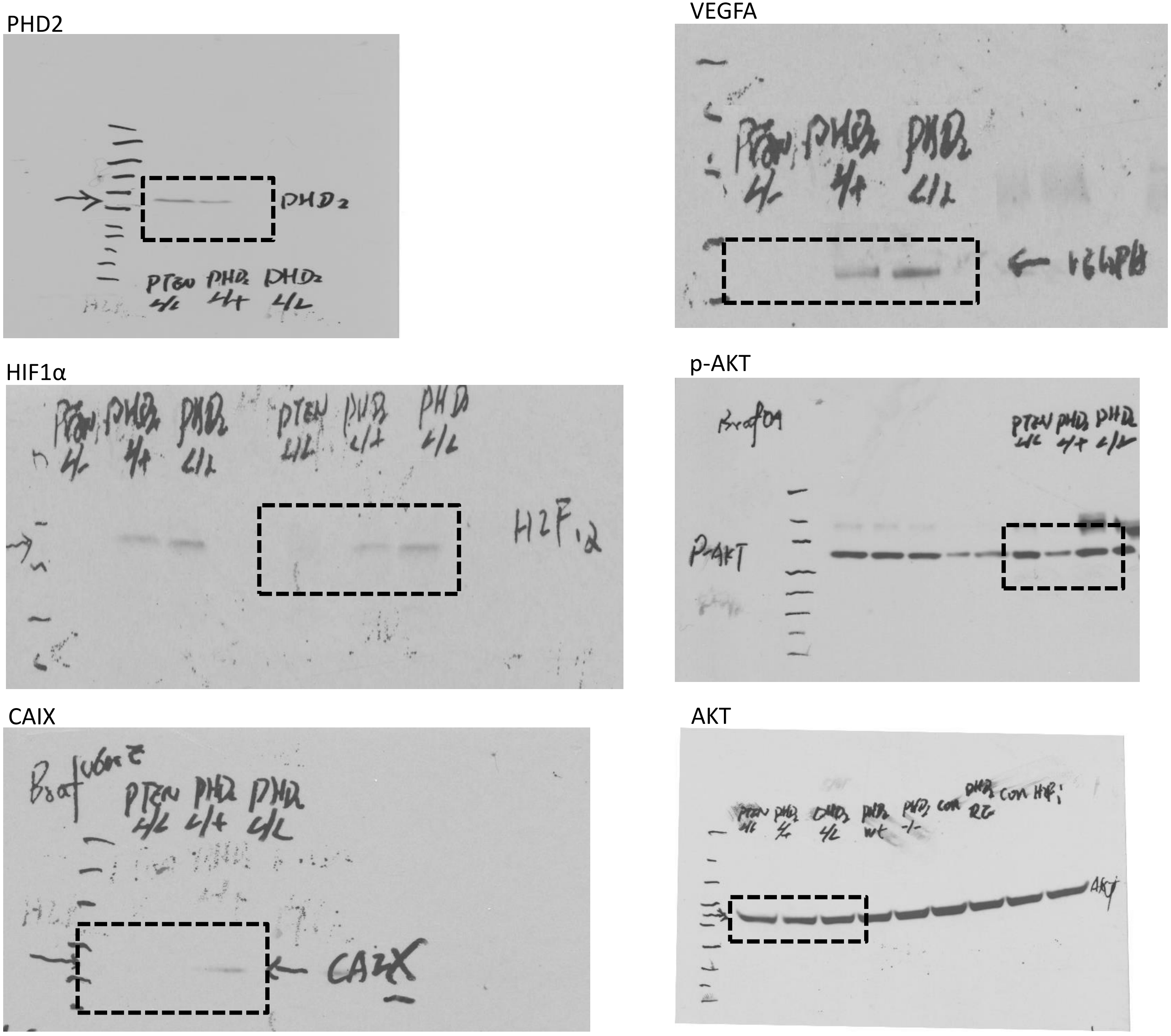

Fig. 5b

β-actin

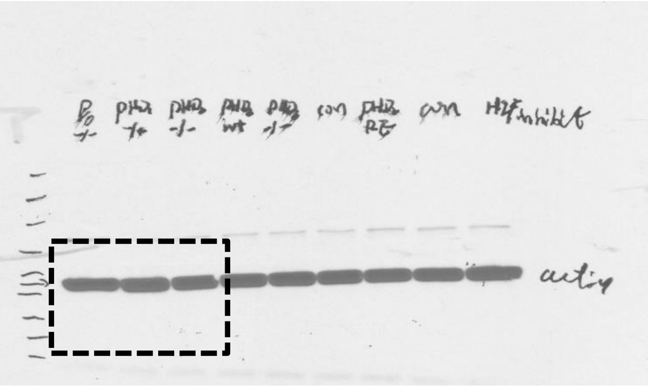

Fig. 6b

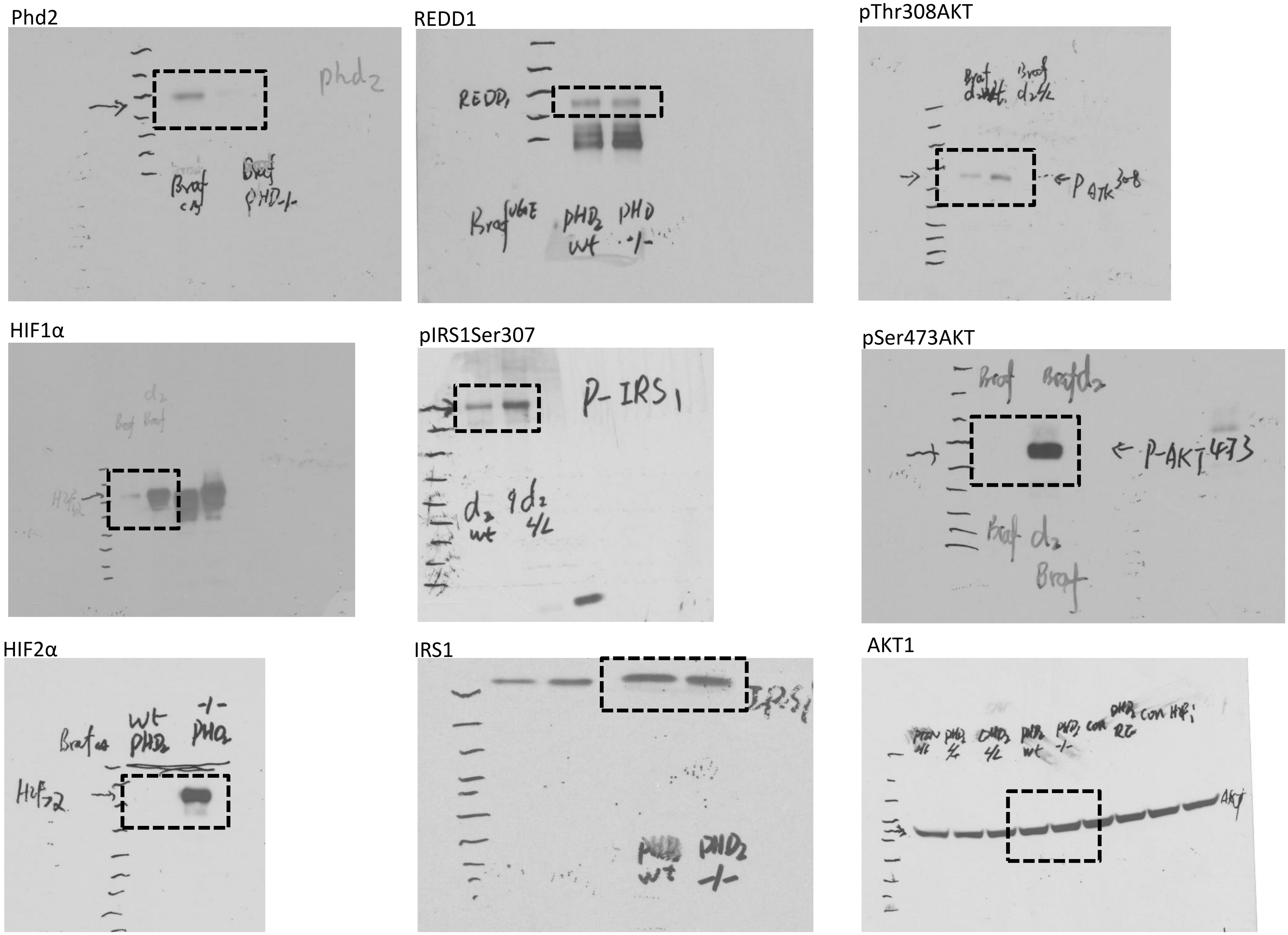
Phd2
REDD1
pThr308AKT
HIF1α
pIRS1Ser307
pSer473AKT
HIF2α
IRS1
AKT1

Fig. 6b

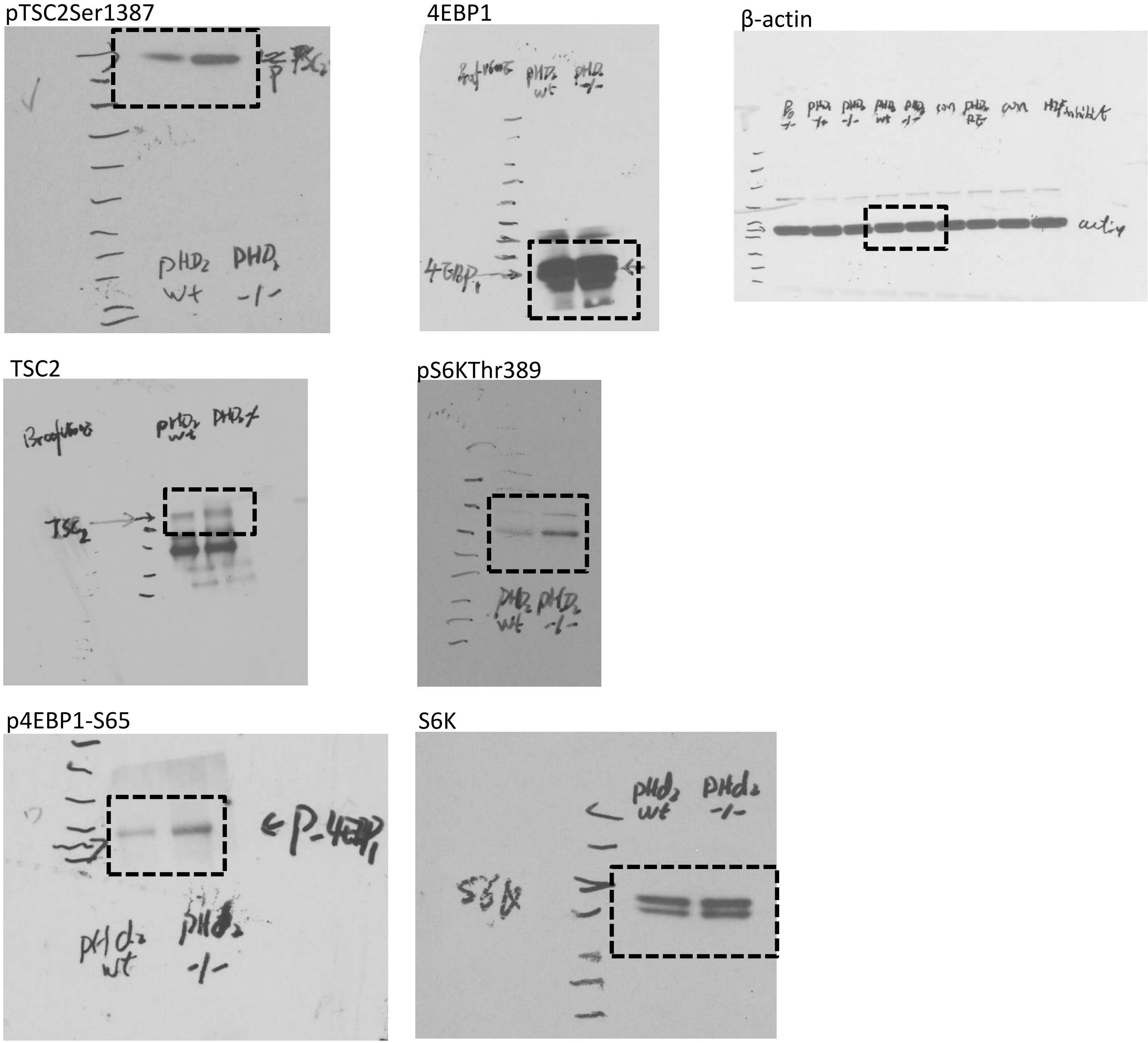

Fig. 6c

Phd2

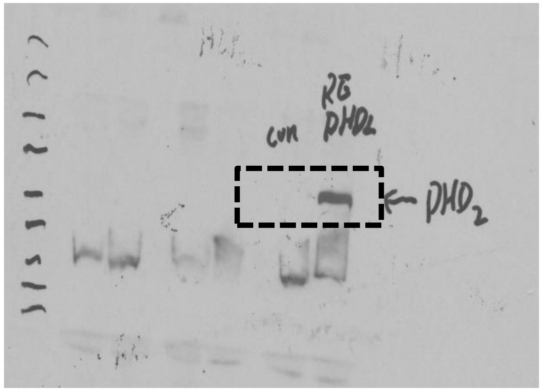

HIF1α

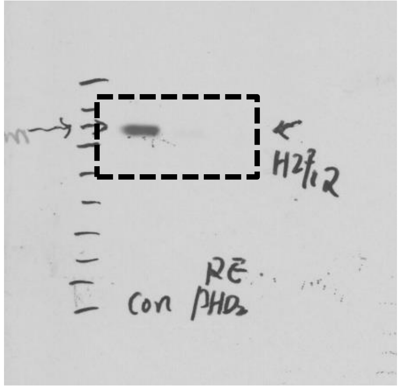

HIF2α

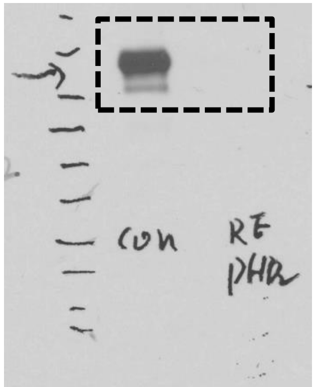

Fig. 6c

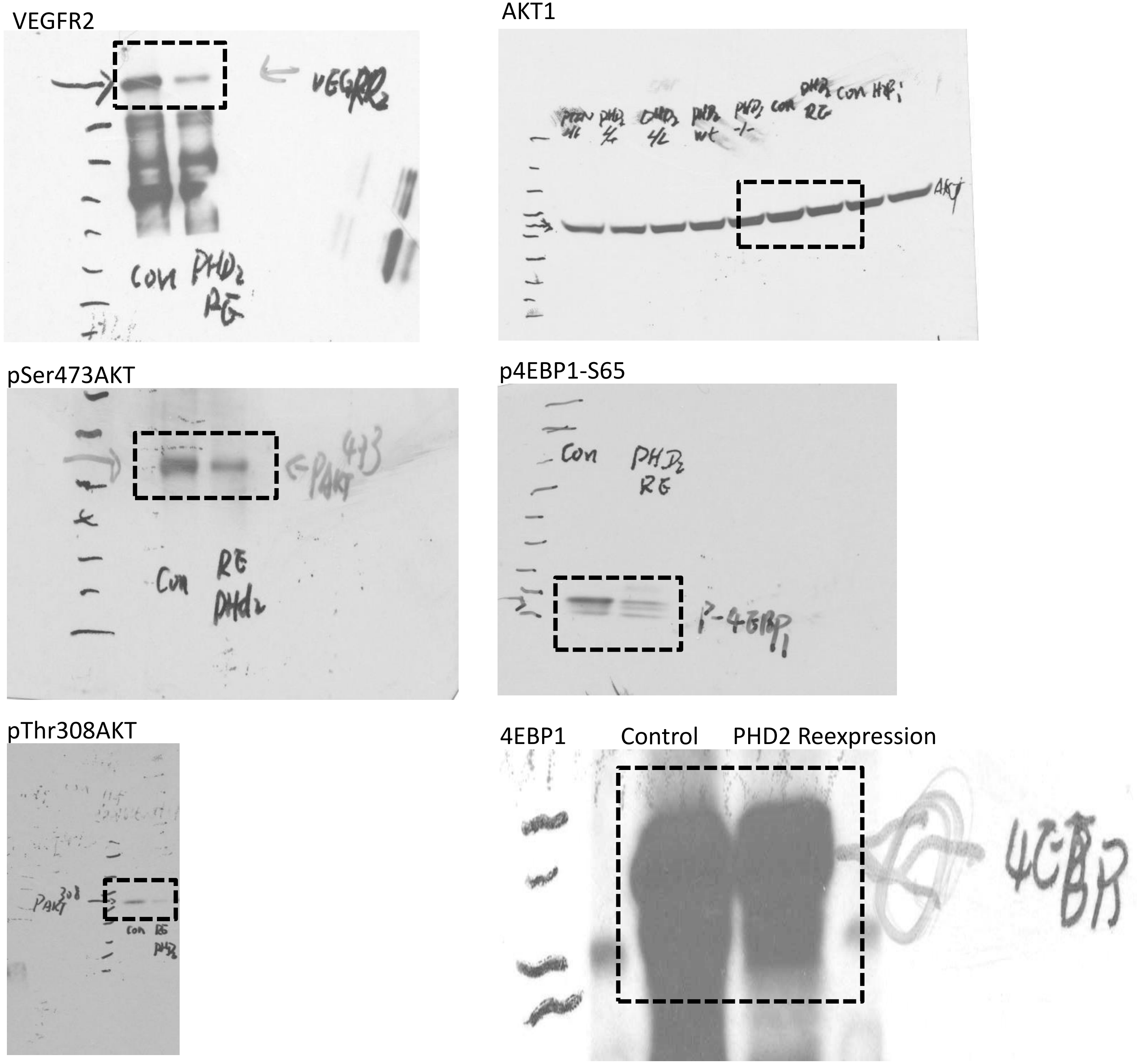
VEGFR2
AKT1
pSer473AKT
p4EBP1-S65
pThr308AKT
4EBP1
Control
PHD2 Reexpression

Fig. 6c

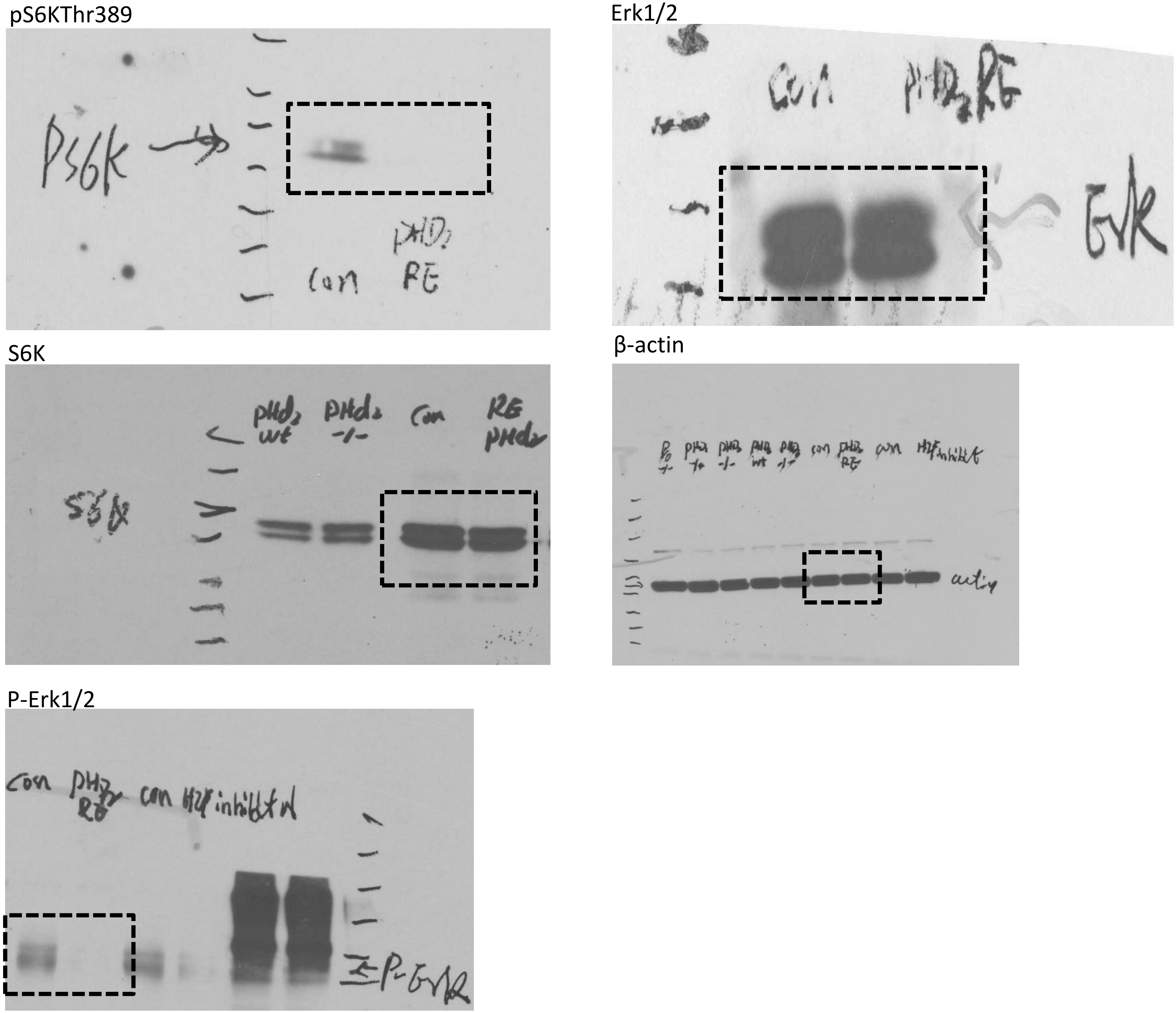

Fig. 6d

HIF1α

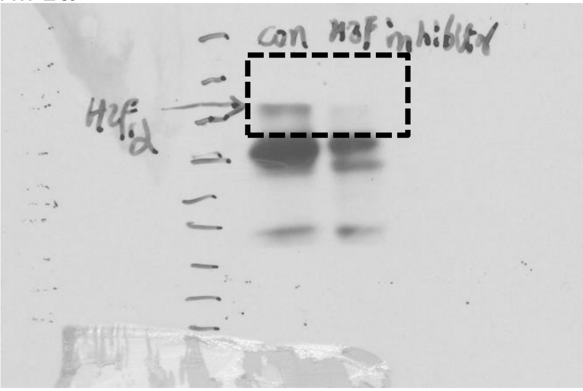

pSer473AKT

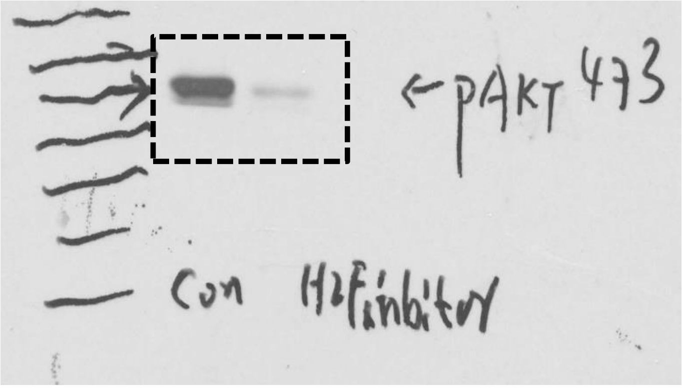

HIF2α

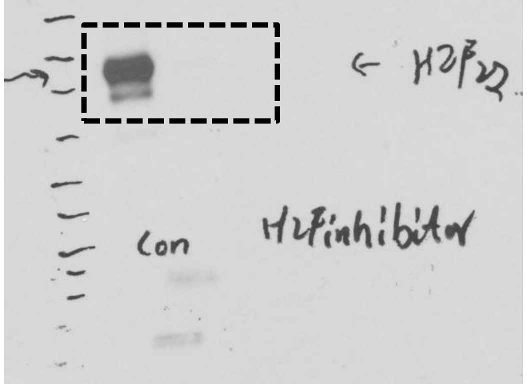

pThr308AKT

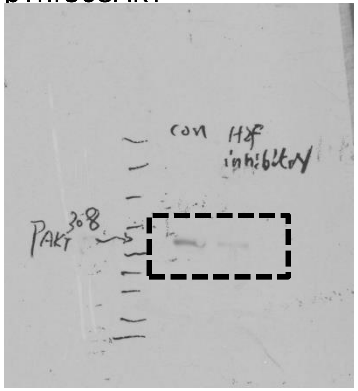

VEGFR2

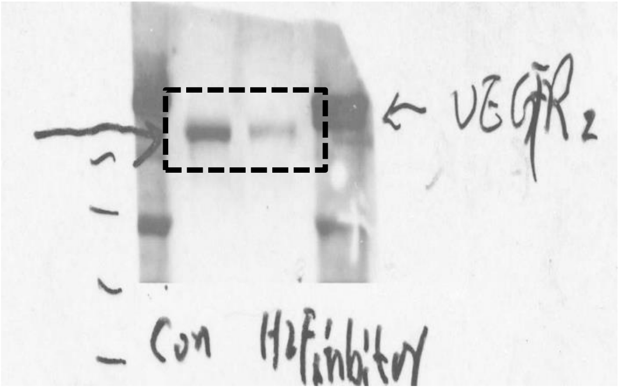

AKT1

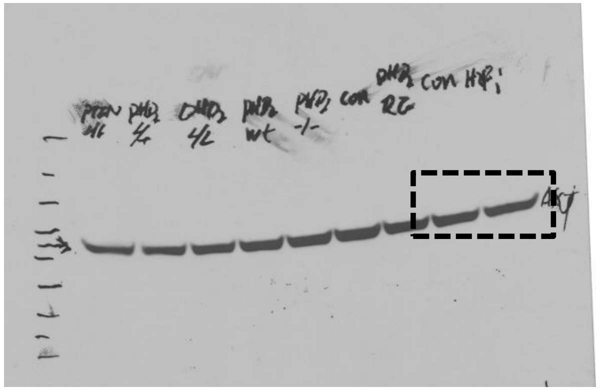

Fig. 6d

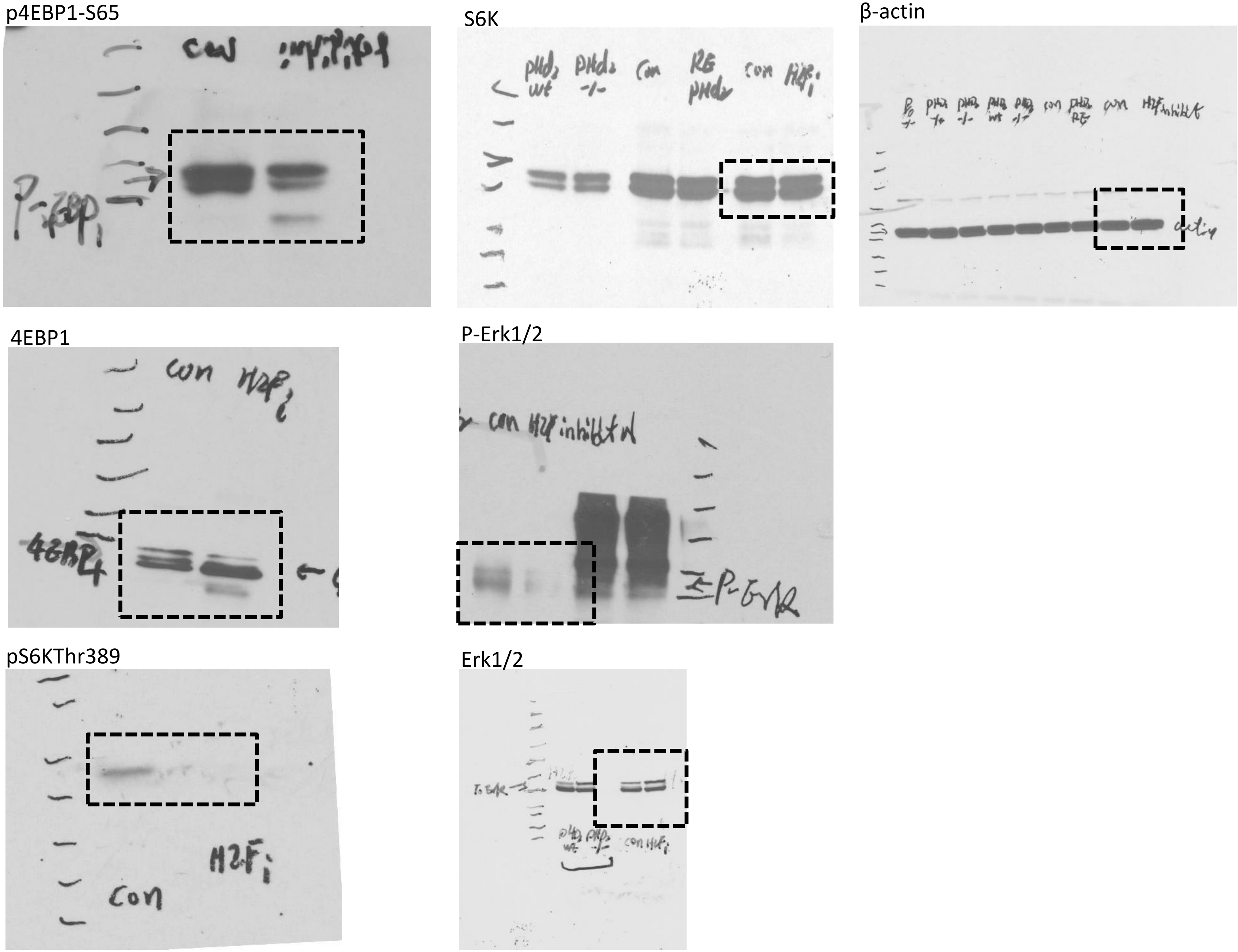

Fig. 7k

VEGFR2

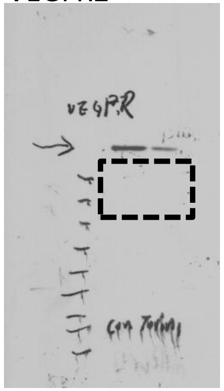

AKT1

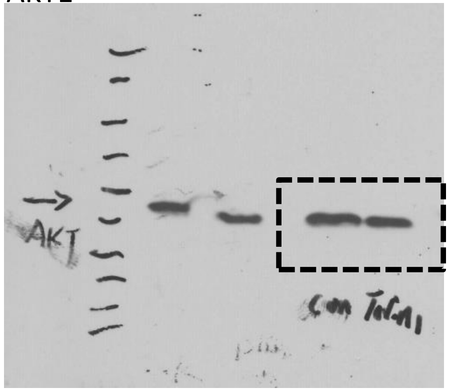

P-Erk1/2

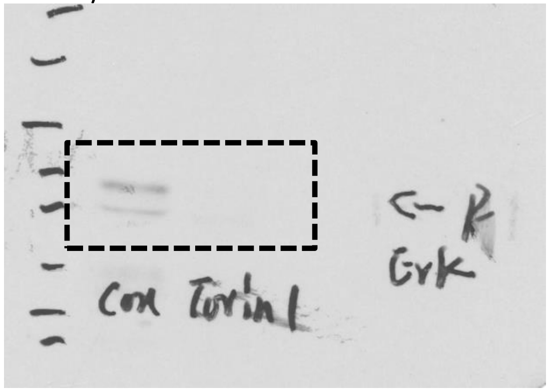

pSer473AKT

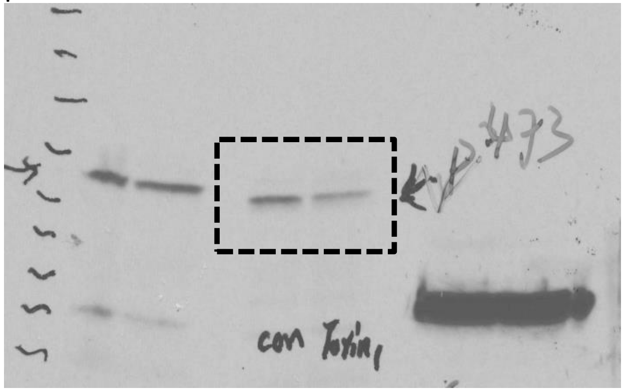

pS6KThr389

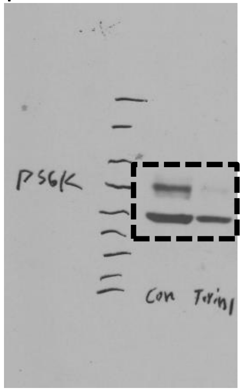

Erk1/2

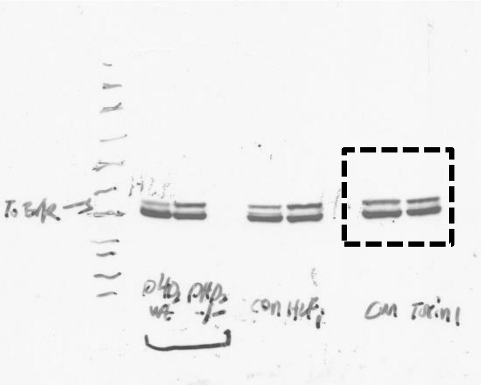

pThr308AKT

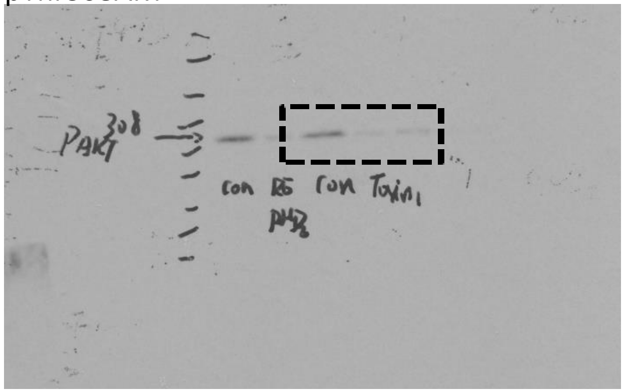

S6K

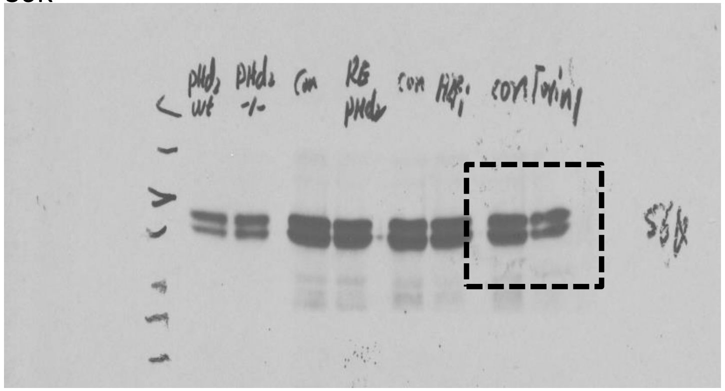

β-actin

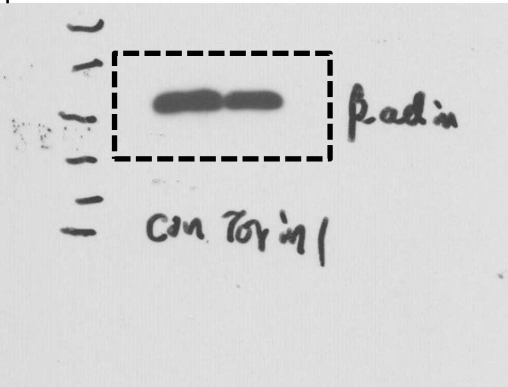

Supplementary Figure 10. Full immunoblots.
